# Physiological responses in sea trout to repeated salmon louse infections and freshwater

**DOI:** 10.1093/conphys/coaf080

**Published:** 2025-11-25

**Authors:** Per Gunnar Fjelldal, Sussie Dalvin, Christine Sørfonn, Bjørnar Skjold, Audun Østby Pedersen, Tom J Hansen, Ørjan Karlsen

**Affiliations:** Reproduction and Developmental Biology, Institute of Marine Research (IMR), Matre Research Station, 5984 Matredal, Norway; Pathogen Transmission and Disease, Institute of Marine Research (IMR), PO Box 1870, Nordnes, 5817 Bergen, Norway; Reproduction and Developmental Biology, Institute of Marine Research (IMR), Matre Research Station, 5984 Matredal, Norway; Pathogen Transmission and Disease, Institute of Marine Research (IMR), PO Box 1870, Nordnes, 5817 Bergen, Norway; Reproduction and Developmental Biology, Institute of Marine Research (IMR), Matre Research Station, 5984 Matredal, Norway; Reproduction and Developmental Biology, Institute of Marine Research (IMR), Matre Research Station, 5984 Matredal, Norway; Reproduction and Developmental Biology, Institute of Marine Research (IMR), PO Box 1870, Nordnes, 5817 Bergen, Norway

**Keywords:** Acid–base regulation, osmoregulation, physiology, salmon louse, sea trout

## Abstract

Sea trout (*Salmo trutta*) migrate to the seawater (SW) for increased food availability. However, heavy infestations with salmon louse (*Lepeophtheirus salmonis*) can make them return to freshwater (FW). The aim of the present study was to map if and how reinfection with salmon louse and repeated FW exposure affects survival, growth rate, hepatosomatic index (HSI), acid base regulation (plasma pH, strong ion difference), osmoregulation (plasma ions, osmolality) and semen quality (fertilization rate, embryo/fry survival) in sea trout. Individually tagged sea trout (~100 g) were infected with louse copepodids in SW and then switched to FW at the louse pre-adult stage. Twelve days thereafter, FW was replaced with SW, and a second similar louse infection and salinity change were performed. Treatment groups were (i) uninfected control, and infected during the first (ii), second (iii) or both (iv) infection periods. The study ended after a final three-month follow-up in FW involving egg fertilization with sperm of previously infected and uninfected control mature male trout.

Lice infection did not affect fish mortality or semen quality, but elevated HSI. In SW, lice-infected fish had lower specific growth rate in weight, higher plasma pH, Na^+^, Cl^−^ and osmolality, and lower plasma strong ionic difference and Na^+^/Cl^−^ ratio compared to uninfected fish. After 48 h in FW, lice-infected fish still had higher plasma pH, while plasma Na^+^, Cl^−^ and osmolality were lower and plasma Na^+^/Cl^−^ ratio higher in infected than uninfected fish. Louse reinfection did not affect any end points compared to single infection.

The results demonstrate that salmon louse disturbs sea trout’s Cl^−^ more than Na^+^ regulation, resulting in reduced hypo-osmotic and hyper-osmotic abilities in SW and FW, respectively. Further, a strong effect of lice on acid–base regulation is evident, shown by elevated plasma pH in both SW and FW.

## Introduction

Anadromous brown trout (*Salmo trutta*), also referred to as sea trout, are born in rivers or brooks, where they return to spawn after a period in seawater (SW). Sea trout is a natural prey for several different predators (Rae, 1973; Valle, 1985; Middlemas *et al.*, 2003; Jepsen *et al.*, 2006; Källo *et al.*, 2020, 2023). Modern humans have also fished sea trout (Ritchie *et al.*, 2016; Mjærum and Mansrud, 2020) and later developed recreational fishing (Liu *et al.*, 2019). However, industrialization and human activities have created several new threats, both in freshwater (FW) and SW. Nowadays, the elevated level of the parasitic salmon louse (*Lepeophtheirus salmonis*) produced on farmed salmon poses a threat to marine growth and survival of sea trout (Thorstad *et al.*, 2015) and is considered the largest negative anthropogenic impact factors on wild sea trout populations in Norway (Thorstad *et al.*, 2019). Sea trout populations in areas with open salmon sea cages in Scotland and Ireland are also negatively impacted by salmon louse (reviewed in Thorstad *et al.*, 2015), dating back to 1990 in Ireland (Tully *et al.*, 1993).

Sea trout can return to FW to rid themselves of lice. Salmon louse is a marine ectoparasitic copepod with eight different developmental stages: nauplii I → nauplii II → copepodid → chalimus I → chalimus II → pre-adult I → pre-adult II → adult (Hamre *et al.*, 2013). They infect the host as copepodids and are sedentary until the pre-adult stage when they become mobile and can move around on the host’s body (Bjørn and Finstad, 1998; Hamre *et al.*, 2013). Mobile lice feed on the fish’s skin and mucus, accompanied by elevated plasma ions (Na^+^, Cl^−^), osmolality and cortisol levels (Birkeland and Jakobsen, 1997; Wells *et al.*, 2006, 2007; Fjelldal *et al.*, 2023), elevated liver size (Fjelldal *et al.*, 2023), reduced growth rate, tissue damage and elevated mortality (reviewed in Thorstad *et al.*, 2015). Attached salmon louse are resilient to low salinity but ultimately dies in FW (Finstad *et al.*, 1995; Wright *et al.*, 2016). Infected sea trout exploit an adaptive strategy to reduce louse burden by returning to FW earlier than usual, a behaviour referred to as premature FW return (Tully *et al.*, 1993; Birkeland, 1996; Birkeland and Jakobsen, 1997; Bjørn *et al.*, 2001; Serra-Llinares *et al.*, 2020). After returning, trout may either remain in FW, re-enter SW or in some cases die in FW (Birkeland, 1996). To date, research on the relationship between marine growth of sea trout and sea louse infection has relied exclusively on scale analysis of wild fish (reviewed in Thorstad *et al.*, 2015). Since premature FW returns alter scale growth patterns, it may act as a confounding factor in such studies. Knowledge about individual fish’s growth rates under controlled settings in SW is thus an important research goal. Consequences of lice reinfection after premature FW returns are another topic that deserves attention. The effect of sea lice infestation on host acid–base regulation remains poorly understood. Current knowledge from salmonids suggests that lice infection and/or premature FW return impact acid–base regulation in sea trout: (i) Lice-infected fish have elevated plasma ion level in SW (Birkeland and Jakobsen, 1997), and movement between water salinities affects plasma ion (Fjelldal *et al.*, 2023) and pH (Maxime *et al.*, 1990, 1991) levels. (ii) Lice-infected fish can have reduced appetite (Fjelldal *et al.*, 2019), which may impact their plasma pH (Payan *et al.*, 1998). (iii) Lice infection increases the host’s plasma cortisol level (Wells *et al.*, 2007), which may be indicative of an acid–base disturbance (Collier, 2007; Gilmour *et al.*, 2011). Changes in acid–base regulation may be orchestrated by changes in the plasma strong ion difference (SID) and the ratio between the concentrations of Na^+^ and Cl^−^ (Maxime *et al.*, 1990). The calculation of SID was established in 1981 by P.A. Stewart as an approach in quantitative acid–base chemistry and is calculated as the difference between the concentrations of strong cations (Na^+^, K^+^, Ca^2+^) and strong anions (Cl^−^, lactate) (reviewed in Rinaldi and De Gaudio, 2005). The plasma concentrations of cations and anions are affected by lice infection and/or changing water salinity (Wells *et al.*, 2007; Fjelldal *et al.*, 2019, 2023; Long *et al.*, 2019). Knowledge on this topic can help to understand how pathophysiology connects to host mortality following lice infection.

Salmon louse infections may compromise host reproductive success. For sea trout, as for other anadromous salmonids, migration to the SW habitat provides access to rich feeding grounds that support energy allocation to both somatic growth and gamete development. In Atlantic salmon, evidence from field studies and modelling indicates that louse infection can lower body condition in maturing fish (Susdorf *et al.*, 2018a) and negatively affect population strength by lowering marine survival, fecundity and age at maturity (Vollset *et al.*, 2014; Susdorf *et al.*, 2018b; Vollset and Krkosek, 2021). Louse infection may also have a direct negative effect on host gamete quality. Sea trout, for example, display elevated blood plasma osmolality following sea louse infection (Fjelldal *et al.*, 2023). Since blood and seminal plasmas have equal osmolality in mature male chum salmon (*Oncorhynchus keta*) (Morisawa *et al.*, 1979) and increased seminal plasma osmolality has been shown to reduce sperm motility in studies in northern pike (*Esox lucius* L.) (Alavi *et al.*, 2009), lice-induced osmoregulatory disturbances may compromise sperm function. Moreover, possible lice-induced changes in acid–base regulation may impede sperm functions (Mishra *et al.*, 2018). As such, salmon louse infection may reduce gamete quality in sexually mature sea trout, which would be an unexplored negative effect of salmon louse on wild sea trout populations. This is vital knowledge for sea trout conservation since adult sea trout may be heavily burdened by lice at river ascendence (Birkeland, 1996).

The aim of the present study was to examine the combined effects of salmon louse infection and reinfection, and subsequent simulated premature FW return on:

1. Growth rates for weight [specific growth rate (SGR)] and length (mm day^−1^).
2. Liver size [hepatosomatic index (HSI)].
3. Osmoregulation (plasma ions osmolality, Na^+^, Cl^−^)
4. Acid–base status (plasma pH, SID Na^+^/Cl^−^)
5. Semen quality (fertilization rate, egg/fry mortality).

## Materials and Methods

### Experimental procedure/rationale

**Figure 1:**
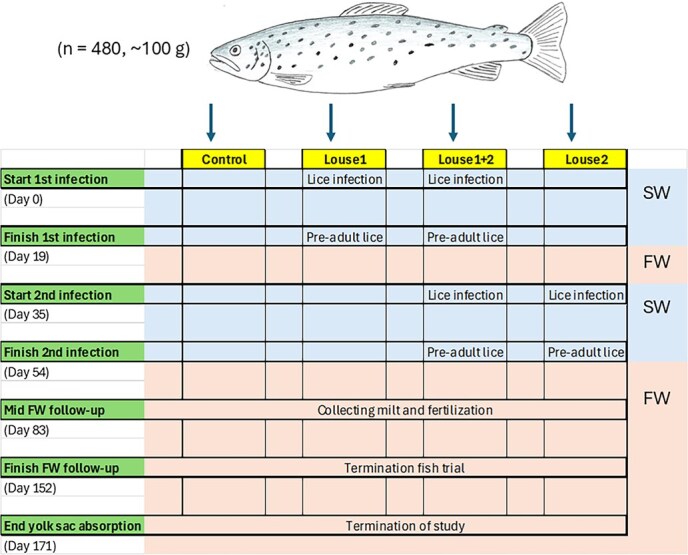
Experimental design. 480 1-year-old PIT-tagged sea trout (*S. trutta*) were used in the study. There were two sampling points at ‘Finish first infection’ and at ‘Finish second infection’; first a sampling in SW followed by a sampling after 48 h in FW. Milt was collected from sexually mature male trout during the FW follow-up for fertilization and evaluation of fertilization rate and embryo/fry survival.

Sea trout maintained in SW for 3 months were divided into four groups: one control group and three groups exposed to infection at different time points. The experiment had two infection periods in SW separated by a 12-day stay in FW (to simulate a premature FW return caused by lice and a subsequent migration to SW after a period in FW) and a final follow-up period in FW after the second period in SW (to simulate a second premature FW return). Among the three different infection groups, one was infected during the first infection period only (Louse1), one was infected during both the first and second infection period (Louse1 + 2), and one was infected during the second infection period only (Louse2). The outline of the experiment is shown in Fig. 1.

The research team previously conducted a similar study on 2-year-old sea trout infected immediately after transfer from FW to SW (Fjelldal *et al.*, 2023). Based on insights into that work regarding osmoregulatory and maturity development, the present study used 1-year-old trout that were maintained in SW for 3 months prior to the first infection. The design was used for two reasons: (i) In the current trout population, most males become sexually mature at 2 years old and females at 3 years old in laboratory studies (authors’ own observations) and (ii) hatchery-reared sea trout needs to stay in SW for a period before all individuals have achieved full hypo-osmoregulatory ability (Jonsson *et al.*, 1994; Fjelldal *et al.*, 2023).

### Fisk stock

One-year-old hatchery-reared sea trout (*S. trutta*) of the Matre River strain reared in 0.8 ppt FW under simulated natural photoperiod and ambient temperature were gradually shifted to 34.0 ppt SW over 16 days from 08 May to 24 May in 2023 and then reared at 34 ppt and 9°C until the experimental started in July 2023. The fish originated from a mixed family group where the eggs from six females were mixed and fertilized with milt from two males on 24 November 2022. The eggs were incubated at 8°C, and the fish were reared at 12°C from first feeding in March 2023 until June when they were switched to natural temperature. The fish were kept under continuous light from first feeding until 01 October and then simulated natural photoperiod thereafter.

### Ethical statement

All experiments were performed at the Institute of Marine Research (IMR), Matre Research Station (60° N, 5° E, Western Norway), which is authorized for animal experimentation (Norwegian Food Safety Authority, facility 110) in accordance with international guidelines. The experiment was performed under research permit number 29086.

### Experimental design

On 11 July 2023, 480 sea trout were PIT tagged (Glass tag 2, 12 mm; TrackID AS, Stavanger, Norway) and distributed in 16 square, white, covered fibreglass tanks (1 × 1 × 0.43 m, 30 fish per tank) under simulated natural photoperiod supplied with 10°C SW (34 ppt). On 18 August, the temperature was increased to 12°C and photoperiod shifted to LD18:6. On 25 August, all fish were anaesthetized (0.01 g l^−1^, Aquacalm vet.; Scan Aqua AS, Årnes, Norway) to measure fork length and body weight and identify the PIT tag.

On 29 August (day 0) (Fig. 1; ‘Start first infection’), eight of the tanks were infected with salmon lice (*L. salmonis*) copepodids (Fig. 1; ‘Louse1’ and ‘Louse1 ± 2’), while eight tanks underwent the same procedures, but no lice were added (Fig. 1; ‘control’ and ‘Louse2’). In all 16 tanks (8 infected, 8 uninfected), the water level was reduced to a depth of 10 cm, and water flow was stopped before adding copepodids (10 days post-hatch) to the eight infection tanks. The water flow (normal) was turned back after 10 min. In total, 20 000 copepodids were used to infect the fish (2500 copepodids per tank), giving an average infection pressure of 83 lice per fish. On 13 September (day 19) (Fig. 1; ‘Finish first infection’), the inlet water changed from SW to FW. All fish remained in FW for the next 12 days. Then, the inlet water was gradually changed from FW to SW between 25 and 29 September.

On 03 October (day 35) (Fig. 1; ‘Start second infection’), eight tanks were infected with salmon lice (four previously infected during the first infection, Louse1 ± 2; four previously uninfected during the first infection, Louse2), while eight tanks were uninfected (four previously infected during the first infection, Louse1; four previously uninfected during the first infection, control). The infection method was the same as during the first infection, but now a total of 17 000 copepodids were used to infect the fish (2125 copepodids per tank), corresponding to an average infection pressure of 118 lice per fish. On 18 October (day 54) (Fig. 1; ‘Finish second infection’), inlet water was shifted from SW to FW.

On 16 November, the rearing conditions were changed from 12°C and LD18:6 to ambient temperature and simulated natural photoperiod. On 20 November (day 83; Fig. 1; ‘Mid FW follow-up’), milt from sexually mature males was used to fertilize eggs from 3-year-old female trout (not from the study population, but same strain). Fertilized eggs were incubated at 8°C, with hatching beginning on 24 December. On 24 January 2024 (day 152; Fig. 1; ‘Finish FW follow-up’), all remaining fish were euthanized and sampled (see next chapter). Finally, on 12 February 2024 (day 171; Fig. 1; ‘End yolk sac absorption’), fry had completed yolk sac absorption and were euthanized and counted.

Dead fish, eggs and fry were removed and counted at all stages of the study to calculate mortality rates. The experiment was performed in a flow-through system where natural FW and SW is filtrated (40 μm) and irradiated with UV light. FW is added to SW to reach a salinity of 0.8 ppt for improved buffer capacity. The oxygen content in rearing water was always above 80% in the present experiment. In the experimental facility, groups of four tanks are connected to the same header tank. To prevent possible systematic biases, there was one tank from each of the four treatment groups (control, Louse1, Louse2, Louse1 + 2) within each such four-tank group.

### Samplings

Fish were sampled four times during the experiment: on 13 (Finish first infection, in SW before change to FW) and 15 September (Finish first infection, 48 h after change to FW), and 18 (Finish second infection, SW) and 20 October (Finish second infection, 48 h FW). Each time, four fish per tank were anaesthetized (0.01 g l^−1^, Aquacalm vet., Scan Aqua AS, Årnes, Norway), sampled for blood and counted for lice followed by euthanasia by a sharp blow to the head. Subsequently, PIT tag was recorded, fork length and body weight were measured, the liver was weighed and sex and maturity status were determined. There were no remaining lice on the fish on 29 September after the first period in FW.

To allow for the calculation of SGR, mm day^−1^ and change in the condition factor of the sampled individuals during the first and second infection periods, and during the FW follow-up, all fish were anaesthetized (0.01 g l^−1^, Aquacalm vet., Scan Aqua AS, Årnes, Norway), recorded for PIT tag and measured for fork length and body weight on 25 August (4 days before ‘Start first infection’), 29 September (4 days before ‘Start second infection’), 20 November (Finish second infection, 48 h FW), 16 November (Mid FW follow-up) and 24 January (Finish FW follow-up). At the final sampling, all the fish were killed by a blow to the head after anaesthesia, and sex and maturity status were determined. Gonads were assessed by weight, gonadosomatic index (GSI) and visual inspection. Of the 457 fish, 113 were classified as mature males, 91 as immature males, whereas all 253 females were immature.

### Plasma analysis

Blood samples were collected from the caudal vessel using a heparinized tuberculin syringe fitted with a 25-gauge needle. Sampling was performed immediately after lice counts and prior to tissue dissection. To expedite the procedure, fish were euthanized by a sharp blow to the head following lice enumeration. Blood was centrifuged and plasma stored at −80°C until analysed. Plasma ions, pH, glucose and lactate levels were detected with an ABL90 FLEX PLUS blood gas analyser (Radiometer Medical ApS, Åkandevej 21, DK-2700, Brønshøj, Denmark). Plasma osmolality was determined by freeze point determination (Fiske microosmometer Model 210, Norwood, MA).

### Calculations and statistical analysis

For data analysis the following parameters were calculated:

Infection intensity = Ln ÷ Fw, where Ln was number of lice on infected fish and Fw was body weight (g) of infected fish at time of counting lice.

Condition factor (CF) = (Fw × 100) ÷ Fl^3^, where Fw was body weight (g) and Fl was fork length (cm).

GSI = (Gw *×* 100) ÷ Fw, where Gw was gonad weight and Fw body weight.

HSI = (Hw × 100) ÷ Fw, where Hw was liver weight and Fw body weight.

SGR = (e^G^ − 1)100, where G = (ln(Fw_2_) − ln(Fw_1_))/(*t*_2_ − *t*_1_), Fw_2_ and Fw_1_ were the body weights at times *t*_2_ and *t*_1_.

Millimetre per day (mm day^−1^): mm day^−1^ = (Fl_2_ − Fl_1_) ÷ (*t*_2_ − *t*_1_), where Fl_2_ and Fl_1_ were the fork lengths at times *t*_2_ and *t*_1_.

Change in CF (ΔCF) = CF_2_ − CF_1_, where CF_1_ was CF on sampling number 1, and CF_2_ was CF on sampling number 2.

SID = ([Na^+^] + [K^+^] + [Ca^2+^]) − ([Cl^−^] + [lactate]).

The data were analyzed using Statistica version 12 (StatSoft, Inc., 2300 East 14th Street, Tulsa, OK). Results are shown as means with their standard errors. Data were tested for homogeneity in variance (Levene’s *F* test). For all test, *P* < 0.05 was considered statistically significant.

Significant differences in infection intensity were tested using a mixed model factorial ANOVA design with the tank as the random factor and fish phenotype as fixed factors at Finish first infection, and with the tank as the random factor and fish phenotype and infection group as fixed factors at Finish second infection. Statistical analysis of mortality was performed by Cox proportional hazards regressions using the survival time model.

Data from the first infection period were analysed with the following treatment groups infected (eight tanks; Louse1 and Louse1 + 2) vs. uninfected (eight tanks; control and Louse2), while data thereafter were analysed with the following treatment groups: Louse1, Louse2, Louse1 + 2 and control (four tanks each).

Fish phenotype (mature male, immature male/female) was included in the analysis of length, weight, CF, mm day^−1^, SGR, ΔCF, HSI and GSI. HIS and GSI data at Finish first and second infection include eight euthanized fish per tank (data from SW and 48 h FW pooled). Length, weight, CF, mm day^−1^, SGR and ΔCF data from Finish first infection are from the same eight euthanized fish per tank, while length, weight, CF, mm day^−1^, SGR and ΔCF data from Finish second infection includes the eight euthanized fish per tank plus all the fish that survived to the final sampling (~8.5 fish per tank). Significant differences in length, weight, CF, mm day^−1^, SGR, ΔCF, HSI and GSI within sampling points were tested using a mixed model factorial ANOVA design with tank as the random factor and fish phenotype and infection group as fixed factors. The model applied tested the interaction effects between the two fixed factors. Significant ANOVAs were followed by Unequal N HSD *post hoc* tests to detect possible differences between infection groups and fish phenotypes.

Fish phenotype was not included in the analysis of plasma parameters since numbers were low and there were no significant effects of fish phenotype. Data on plasma parameters measured at Finish first and second infection include four fish per tank per time point (SW and 48 h SW not pooled). Significant differences in plasma parameters at Finish first and second infection were tested using a mixed model factorial ANOVA design with the tank as the random factor and time point (SW vs. 48 h FW) and infection group as fixed factors. The model applied tested the interaction effects between the two fixed factors. Significant ANOVAs were followed by Student–Keuls *post hoc* tests to defect possible differences between sampling points and infection groups.

Where mixed model factorial ANOVAs were applied, the data were also tested with a nested ANOVA design where the tank was nested in the treatment group as a random factor. Both approaches gave the same significance levels.

To inspect for possible significant relationships between plasma parameters and lice infection intensity, data from infected fish from Finish first and second infection SW sampling point were analyzed together with fish phenotypes pooled (*n* = 64). Possible significant linear regressions were tested by simple linear regressions.

## Results

### Infection success was not affected by repeated infections

Both salmon lice infections were successful, resulting in 100% prevalence among infected fish. At Finish first infection, the mean infection intensity was 0.29 ± 0.03 lice g^−1^ (range, 0.23 and 0.40 lice g^−1^), and at Finish second infection, the mean infection intensity was 0.61 ± 0.06 lice g^−1^ (range, 0.34 and 0.85 lice g^−1^) (Fig. S1). At Finish first and second infection, most lice were at the pre-adult 1 stage with a smaller fraction, 8% and 4%, respectively, at the chalimus 2 stage. At Finish second infection, naïve fish (Louse2) carried a higher number of lice (67 lice fish^−1^, 0.71 lice g^−1^) compared to previously infected fish (Louse1 + 2; 51 lice fish^−1^, 0.51 lice g^−1^), although the difference was not statistically significant. Similarly, no significant differences in infection intensity were detected at the pre-adult stage in SW—neither between fish phenotypes at Finish first infection (*P* = 0.1665, mixed-model ANOVA), nor between fish phenotypes (*P* = 0.2090) or infection groups (*P* = 0.2241) at Finish second infection.

### Lice infection did not cause significant mortality

Survival analysis indicated slightly higher, though not statistically significant, mortality in lice-infested groups compared to the control group (Table 1 and Fig. S2). Cumulative mortality rates were control = 4.67%, Louse1 + 2 = 6.31%, Louse2 = 8.04%, Louse1 = 9.58%. In total, 21 fish died during the study period.

**Table 1:** Survival analysis using Cox proportional hazards regression (the control group served as reference)

Level of effect	Parameter estimate	Standard error	*P*-value	Hazard ratio	95% Hazard ratio lower CL	95% Hazard ratio upper CL
Louse1	0.7455	0.7072	0.2918	2.1074	0.5270	8.4280
Louse2	0.5613	0.7303	0.4421	1.7530	0.4189	7.3360
Louse1 + 2	0.3106	0.7638	0.6843	1.3642	0.3053	6.0956

CL, confidence limit.

### Lice infection did not affect fish length or weight

There were no significant effects of infection group or fish phenotype on length or weight at any sampling points (mixed model ANOVA, *P* > 0.05; Table S1). The condition factor, however, was significantly affected by fish phenotype at all sampling points except the terminal sampling point at Finish FW follow-up (mixed model ANOVA, *P* < 0.05). In general, mature males exhibited the highest condition factor at these sampling points (Table S1).

### Lice infection reduced SGR in SW

SGR was significantly affected by infection group during both infection periods and by phenotype during the last period of the FW follow-up (mixed model ANOVA, *P* < 0.05; Table 2). During the first infection period, uninfected females (control and Louse2 pooled) exhibited significantly higher SGR than infected females (Louse1 and Louse1 + 2 pooled). During the second infection period, Louse1 + 2 and Louse2 females had significantly lower SGR compared to control and Louse1 females. During the last period of the FW follow-up, mature males showed significantly lower SGR compared to immature males and females within all four infection groups, and females had significantly lower SGR compared to immature males within the Louse2 group.

**Table 2:** Mean ± SE calculated SGR (mm day^−1^) and delta condition factor for individually tagged trout

Time point	Parameter	Control			Louse1			Louse2			Louse1 + 2			*P*-value	
		IM ♂	M ♂	IM ♀	IM ♂	M ♂	IM ♀	IM ♂	M ♂	IM ♀	IM ♂	M ♂	IM ♀	Phenotype	Treatment
First infection period	SGR	0.69 ± 0.08^a,b^	0.81 ± 0.06^a,b^	0.74 ± 0.05^a^	0.48 ± 0.14^a,b^	0.61 ± 0.08^a,b^	0.51 ± 0.05^b^							0.346060	***0.005072***
(Day 0–19)	mm day^−1^	0.42 ± 0.04	0.38 ± 0.03	0.4 ± 0.02	0.3 ± 0.03	0.32 ± 0.04	0.35 ± 0.02							0.667089	0.053631
	ΔCF	0.02 ± 0.02	0.07 ± 0.01	0.04 ± 0.01	0.01 ± 0.04	0.04 ± 0.02	−0.01 ± 0.01							0.070730	0.091349
Second infection period	SGR	0.43 ± 0.04^a,b^	0.35 ± 0.05^a,b^	0.35 ± 0.04^a^	0.27 ± 0.16^a,b^	0.4 ± 0.06^a,b^	0.33 ± 0.04^a^	0.09 ± 0.11 ab	0.2 ± 0.05^a,b^	0 ± 0.05^b^	0.08 ± 0.11^a,b^	0.16 ± 0.07^a,b^	0.03 ± 0.06^b^	0.051715	***0.001455***
(Day 35–54)	mm day^−1^	0.51 ± 0.07	0.5 ± 0.06	0.45 ± 0.04	0.34 ± 0.09	0.47 ± 0.07	0.41 ± 0.04	0.33 ± 0.07	0.39 ± 0.05	0.31 ± 0.03	0.32 ± 0.05	0.29 ± 0.04	0.3 ± 0.03	0.649656	***0.007802***
	ΔCF	−0.08 ± 0.02^c^	−0.1 ± 0.02^a,b,c^	−0.07 ± 0.01^a,b,c^	−0.06 ± 0.02^b,c^	−0.07 ± 0.02^a,b,c^	−0.07 ± 0.02^a,b,c^	−0.1 ± 0.03^b,c^	−0.11 ± 0.03^a,b^	−0.11 ± 0.02^a,b,c^	−0.09 ± 0.03^a,b,c^	−0.08 ± 0.02^a^	−0.1 ± 0.01^a,b^	0.929902	***0.013333***
FW follow-up period 1	SGR	0.61 ± 0.14	0.65 ± 0.15	0.41 ± 0.09	0.47 ± 0.28	0.78 ± 0.07	0.6 ± 0.1	0.87 ± 0.17	0.84 ± 0.09	0.65 ± 0.08	0.69 ± 0.18	0.74 ± 0.1	0.67 ± 0.1	0.129610	0.579070
(Day 54–83)	mm day^−1^	0.43 ± 0.08	0.45 ± 0.09	0.38 ± 0.05	0.38 ± 0.11	0.45 ± 0.09	0.36 ± 0.06	0.46 ± 0.08	0.44 ± 0.06	0.41 ± 0.05	0.42 ± 0.09	0.37 ± 0.04	0.44 ± 0.04	0.799639	0.964414
	ΔCF	0 ± 0.02^a,b^	0.02 ± 0.03^a,b^	−0.03 ± 0.01^b^	−0.01 ± 0.05^a,b^	0.08 ± 0.02^a,b^	0.02 ± 0.02^a,b^	0.06 ± 0.03^a,b^	0.09 ± 0.04^a^	0.03 ± 0.01^a,b^	0.04 ± 0.03^a,b^	0.08 ± 0.02^a,b^	0.02 ± 0.02^a,b^	***0.020868***	0.126661
FW follow-up period 2	SGR	0.19 ± 0.03^a,b^	−0.02 ± 0.03^c^	0.16 ± 0.02^a,b^	0.2 ± 0.03^a,b^	−0.04 ± 0.02^c^	0.18 ± 0.01^a,b^	0.29 ± 0.02^a^	−0.05 ± 0.03^c^	0.14 ± 0.02^b^	0.18 ± 0.02^a,b^	0.03 ± 0.03^c^	0.14 ± 0.01^b^	***0.000000***	0.959194
(Day 83–152)	mm day^−1^	0.13 ± 0.01^a,b^	0.05 ± 0.01^c,d^	0.1 ± 0.01^a,b,c^	0.11 ± 0.01^a,b,c^	0.04 ± 0.01^d^	0.11 ±v 0.01^a,b^	0.14 ± 0.01^a^	0.06 ± 0.01^c,d^	0.11 ± 0.01^a,b^	0.12 ± 0.01^a,b^	0.07 ± 0.01^b,c,d^	0.11 ± 0.01^a,b^	***0.000001***	0.643319
	ΔCF	0.01 ± 0.02^a,b,c^	−0.09 ± 0.02^c,d^	0.03 ± 0.01^a^	0.04 ± 0.02^a^	−0.09 ± 0.02^b,c,d^	0.02 ± 0.01^a^	0.08 ± 0.02^a^	−0.13 ± 0.03^d^	−0.01 ± 0.01^a,b,c^	0.01 ± 0.01^a,b^	−0.07 ± 0.02^b,c,d^	−0.02 ± 0.01^a,b,c^	***0.000000***	0.703210

Number in italic and bold indicates a significant difference (*P* < 0.05) between phenotypes or treatments using a mixed model factorial ANOVA. Different lowercase letters indicate significant differences.

Growth in length, measured as millimetres per day, was significantly influenced by infection group during the second infection period and by phenotype during the last period of the FW follow-up (mixed model ANOVA, *P* < 0.05; Table 2). There were no significant differences in growth in length between fish phenotypes within infection groups or between infection groups within the same phenotype during the second infection period. During the last period of the FW follow-up, mature males grew significantly slower in length than immature males and females within the Louse1 and Louse2 groups, and also grew more slowly than immature males within the control group.

Changes in condition factor (ΔCF) were significantly affected by infection group during the second infection period and by fish phenotype during the FW follow-up periods (mixed model ANOVA, *P* < 0.05; Table 2). There were no significant differences in ΔCF between fish phenotypes within infection groups or within fish phenotypes between infection groups during the second infection period or during the first period of the FW follow-up. However, during the last period of the FW follow-up, mature males had significantly lower ΔCF compared to immature males and females in the Louse1 and Louse2 groups, and compared to immature females in the control group.

### Lice infection elevated HSI

**Figure 2:**
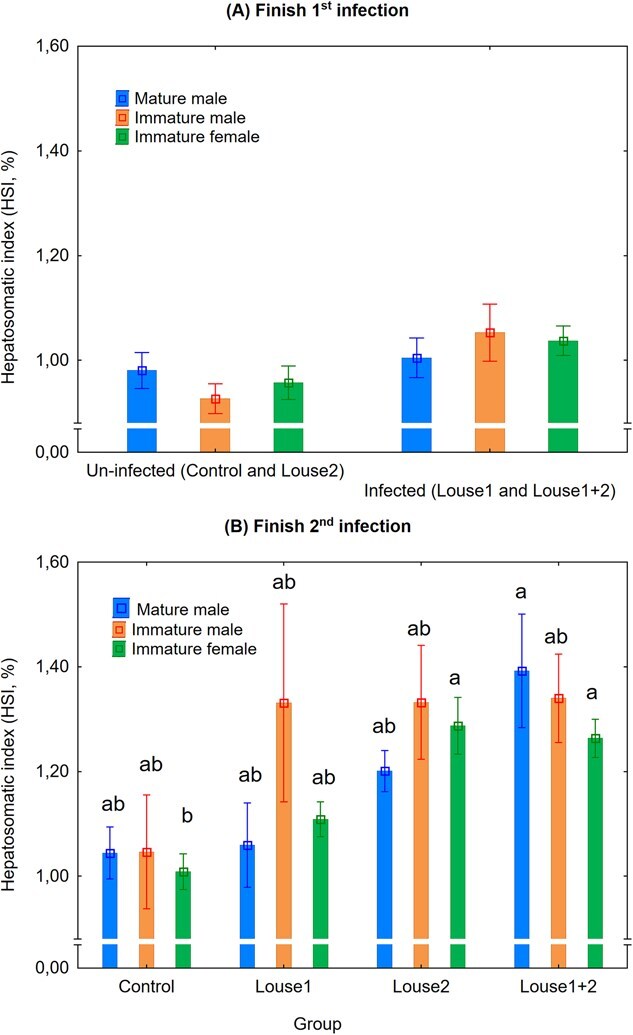
HIS (%, mean ± SE) at Finish first (**A**) and second (**B**) infection (SW and 48 h FW pooled, total *n* per time point = 128; eight per tank). Eight tanks with infected fish (Louse1 and Louse1 + 2) vs. eight tanks with uninfected fish (control and Louse2) at Finish first infection, and four tanks per group at Finish second infection. Different lowercase letters denote significant difference (*P* < 0.05) within each infection period.

There was a significant positive effect (mixed model ANOVA, *P* < 0.05) of lice infection on HSI at Finish first infection, but there were no differences (Unequal N HSD Post-hoc test, *P* > 0.05) between fish phenotypes within infection groups or within fish phenotypes between infection groups (Fig. 2A). Likewise, there was a significant effect of infection group on HSI at Finish second infection (mixed model ANOVA, *P* < 0.01). However, here *post hoc* analysis showed that Louse2 and Louse1 + 2 immature female trout had significantly higher (Unequal N HSD *Post hoc* test, *P* > 0.05) HIS compared to control immature female trout (Fig. 2B).

### Lice infection altered acid–base regulation and reduced hypo-osmotic and hyper-osmotic abilities

**Figure 3:**
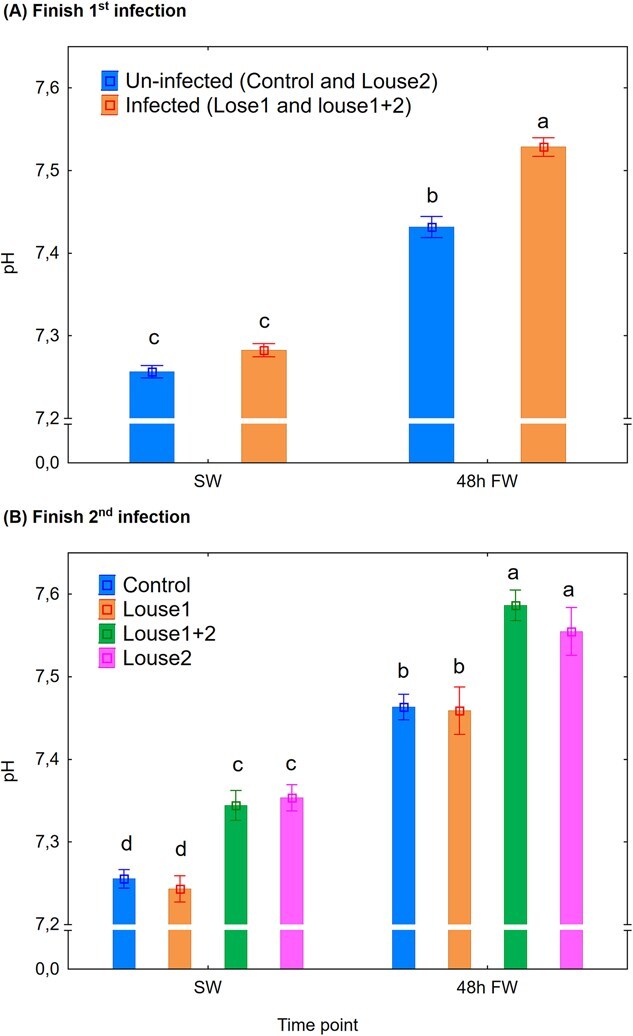
Plasma pH (mean ± SE). (**A**) Finish first infection. (**B**) Finish second infection. SW = at lice pre-adult stage in SW, 48 h FW = 48 h after change to FW. Different lowercase letters denote significant difference (*P* < 0.05) within each infection period.

pH: At Finish first infection, there were significant effects of infection group (mixed model ANOVA, *P* < 0.001) and time point (*P* < 0.001) on plasma pH level. Shift to FW significantly elevated plasma pH, and lice-infected trout had significantly higher (Newman–Keuls *post hoc* test, *P* < 0.05) plasma pH than uninfected trout after 48 h in FW (Fig. 3A). At Finish second infection, there were also significant effects of infection group (*P* < 0.001) and time point (*P* < 0.001) on plasma pH. The control and Louse1 groups had significantly lower plasma pH than the Louse2 and Louse1 + 2 groups both in SW and 48 h FW, and shift to FW significantly elevated plasma pH in all groups (Fig. 3B).

**Figure 4:**
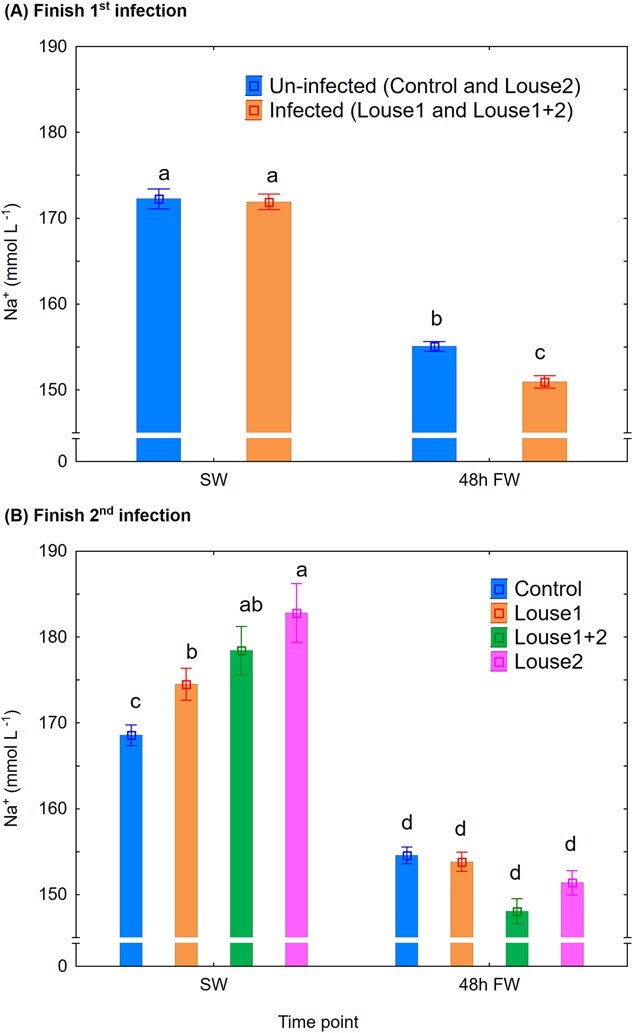
Plasma Na^+^ (mean ± SE). (**A**) Finish first infection. (**B**) Finish second infection. SW = at lice pre-adult stage in SW, 48 h FW = 48 h after change to FW. Different lowercase letters denote significant difference (*P* < 0.05) within each infection period.

Na^+^: At Finish first infection, there were significant effects of both infection group (mixed model ANOVA, *P* = 0.01) and time point (*P* < 0.001) on plasma Na^+^ levels. Here, shift to FW significantly reduced plasma Na^+^, and uninfected fish had significantly higher (Newman–Keuls *post hoc* test, *P* < 0.05) plasma Na^+^ than infected fish after 48 h in FW (Fig. 4A). At Finish second infection, in SW, the control group had significantly lower plasma Na^+^ than the other groups, the Louse1 group (only infected during the previous SW phase) was significantly lower than the Louse2 group, while the Louse1 + 2 group was intermediate between the Louse1 and Louse2 groups. The following shift to FW significantly reduced plasma Na^+^ (mixed model ANOVA, *P* < 0.001, Newman–Keuls *post hoc* test, *P* < 0.05) in all groups (Fig. 4B).

**Figure 5:**
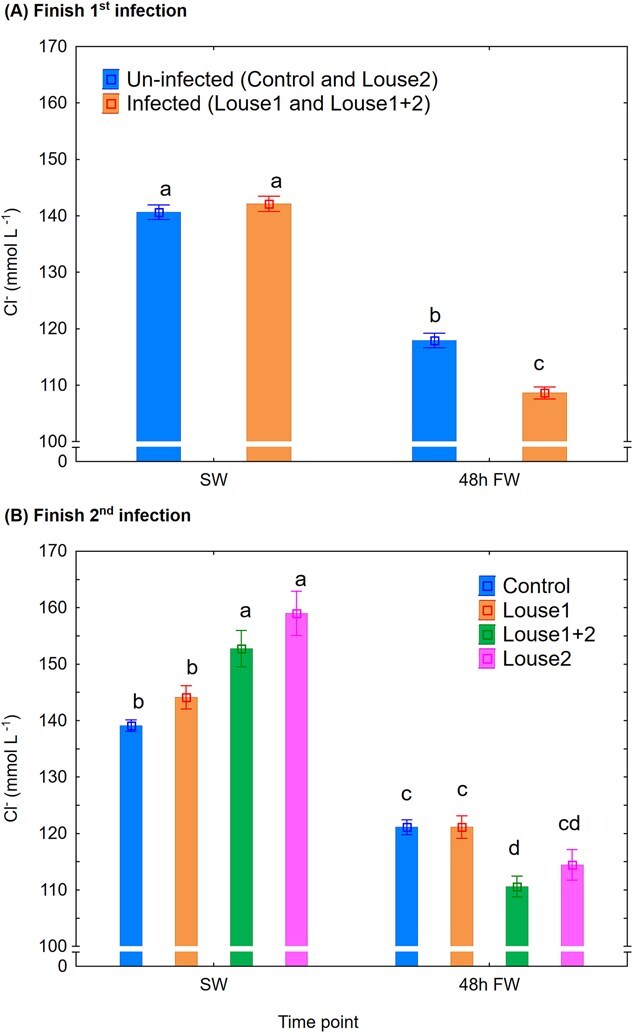
Plasma Cl^−^ (mean ± SE). (**A**) Finish first infection. (**B**) Finish second infection. SW = at lice pre-adult stage in SW, 48 h FW = 48 h after change to FW. Different lowercase letters denote significant difference (*P* < 0.05) within each infection period.

Cl^−^: At Finish first infection, shift to FW significantly reduced (mixed model ANOVA, *P* < 0.001, Newman–Keuls *post hoc* test, *P* < 0.05) plasma Cl^−^, and infected trout had significantly lower levels than uninfected after 48 h in FW (Fig. 5A). At Finish second infection, in SW, the control and Louse1 groups had significantly lower (Newman–Keuls *post hoc* test, *P* < 0.05) plasma Cl^−^ than the Louse2 and Louse1 + 2 groups. The following shift to FW significantly reduced (mixed model ANOVA, *P* < 0.001, Newman–Keuls *post hoc* test, *P* < 0.05) plasma Cl^−^, and the Louse1 + 2 group was significantly lower than the control and Louse1 group after 48 h in FW (Fig. 5B).

**Figure 6:**
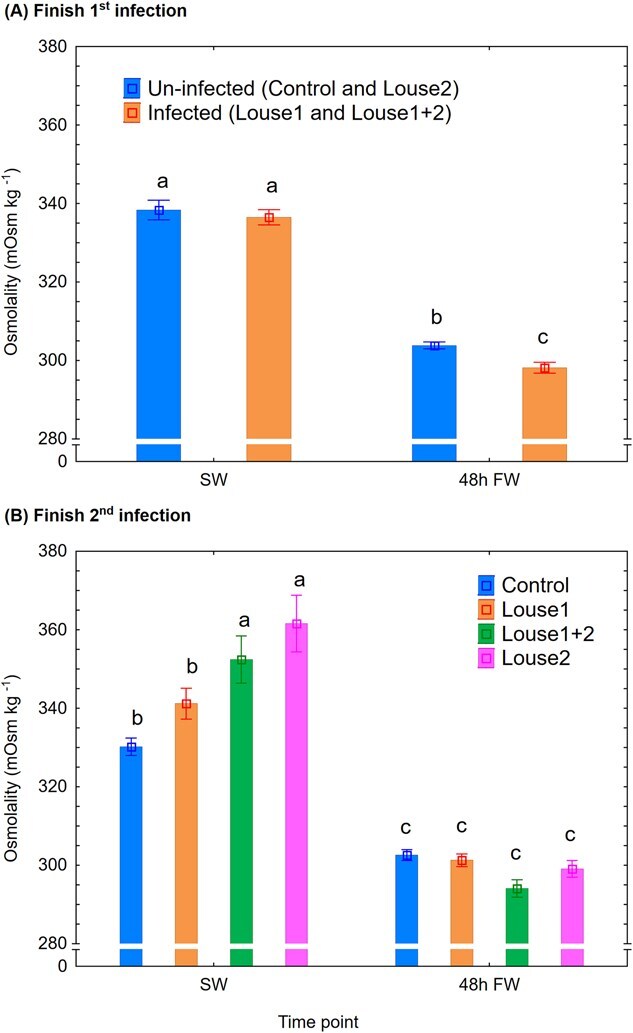
Plasma osmolality (mean ± SE). (**A**) Finish first infection. (**B**) Finish second infection. SW = at lice pre-adult stage in SW, 48 h FW = 48 h after change to FW. Different lowercase letters denote significant difference (*P* < 0.05) within each infection period.

Osmolality: At Finish first infection, infection group (mixed model ANOVA, *P* < 0.05) and time point (*P* < 0.001) had significant effects on plasma osmolality, and infected trout had significantly lower levels (Newman–Keuls *post hoc* test, *P* < 0.05) than uninfected trout after 48 h in FW (Fig. 6A). At Finish second infection, the control and Louse1 groups had significantly lower plasma osmolality than the Louse2 and Louse1 + 2 groups while in SW, and the following shift to FW significantly reduced plasma osmolality (mixed model ANOVA, *P* < 0.001, Newman–Keuls *post hoc* test, *P* < 0.05) (Fig. 6B).

K^+^: At Finish first infection, time point had a significant effect on plasma K^+^ (mixed model ANOVA, *P* = 0.01), and both infected and uninfected fish had significantly reduced (Newman–Keuls *post hoc* test, *P* < 0.05) levels after 48 h in FW (Fig. S3A). At Finish second infection, time point (mixed model ANOVA, *P* < 0.001) and the infection group × time point interaction (*P* = 0.01) had a significant effect on plasma K^+^, and all groups had significantly reduced levels (Newman–Keuls *post hoc* test, *P* < 0.05) following shift to FW at which point the control and Louse1 groups had significantly higher levels than the Louse1 + 2 and Louse2 groups (Fig. S3B).

Ca^2+^: At Finish first infection, time point had a significant effect (mixed model ANOVA, *P* = 0.001) on plasma Ca^2+^, and both infected and uninfected fish had significantly reduced (Newman–Keuls *post hoc* test, *P* < 0.05) levels after 48 h in FW (Fig. S4A). At Finish second infection, there was a significant effect of time point (mixed model ANOVA, *P* < 0.001), infection group (*P* < 0.05) and the interaction between infection group and time point (*P* < 0.05) on plasma Ca^2+^, and the Louse2 group had significantly higher (Newman–Keuls *post hoc* test, *P* < 0.05) levels than the other three groups in SW, and the Louse1 + 2 and Louse2 groups had significantly reduced levels following the shift to FW (Fig. S4B).

Glucose: At Finish first infection, time point had a significant effect (mixed model ANOVA, *P* < 0.05) on plasma glucose, and infected fish had significantly elevated (Newman–Keuls *post hoc* test, *P* < 0.05) levels after shift to FW (Fig. S5). At Finish second infection, there were significant effects of time point (mixed model ANOVA, *P* < 0.05) and the infection group × time point interaction (*P* < 0.05) on plasma glucose, and the Louse1 group had significantly higher (Newman–Keuls *post hoc* test, *P* < 0.05) level than the Louse1 + 2 group in SW, and the Louse1 + 2 group had a significantly increased level following shift to FW (Fig. S5B).

Lactate: At Finish first infection, there was a significant infection group × time point interaction on plasma lactate (mixed model ANOVA, *P* < 0.05), and the infected fish showed significantly elevated levels following shift to FW (Fig. S6A). There were no significant effects on plasma lactate at Finish second infection period.

**Figure 7:**
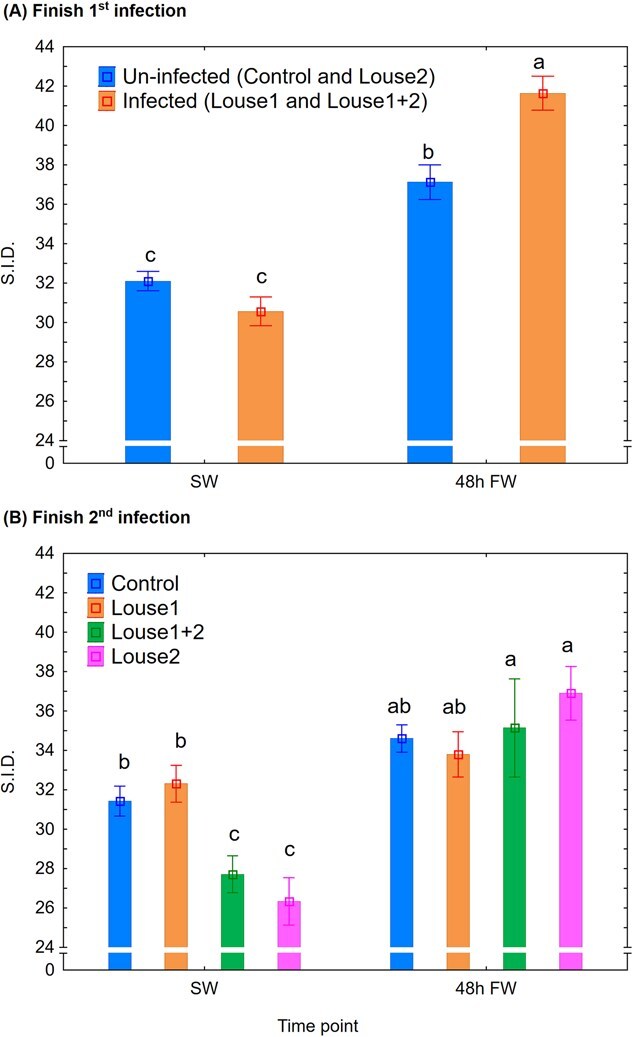
Plasma SID (mean ± SE). (**A**) Finish first infection. (**B**) Finish second infection. SW = at lice pre-adult stage in SW, 48 h FW = 48 h after change to FW. Different lowercase letters denote significant difference (*P* < 0.05) within each infection period.

SID: At Finish first infection, there were significant effects of time point (mixed model ANOVA, *P* < 0.001) and the infection group × time point interaction (*P* < 0.001) on plasma SID, and both infected and uninfected fish had significantly increased SID (Newman–Keuls *post hoc* test, *P* < 0.05) by shift to FW at which time point SID was significantly higher in infected than uninfected fish (Fig. 7A). At Finish second infection period, there were significant effects of time point (mixed model ANOVA, *P* < 0.001) and the infection group × time point interaction (*P* < 0.01) on plasma SID, and the Louse1 + 2 and Louse2 groups had significantly lower SID than the control and Louse1 group in SW (Newman–Keuls *post hoc* test, *P* < 0.05) and also a significant increase in SID following shift to FW (Fig. 7B).

**Figure 8:**
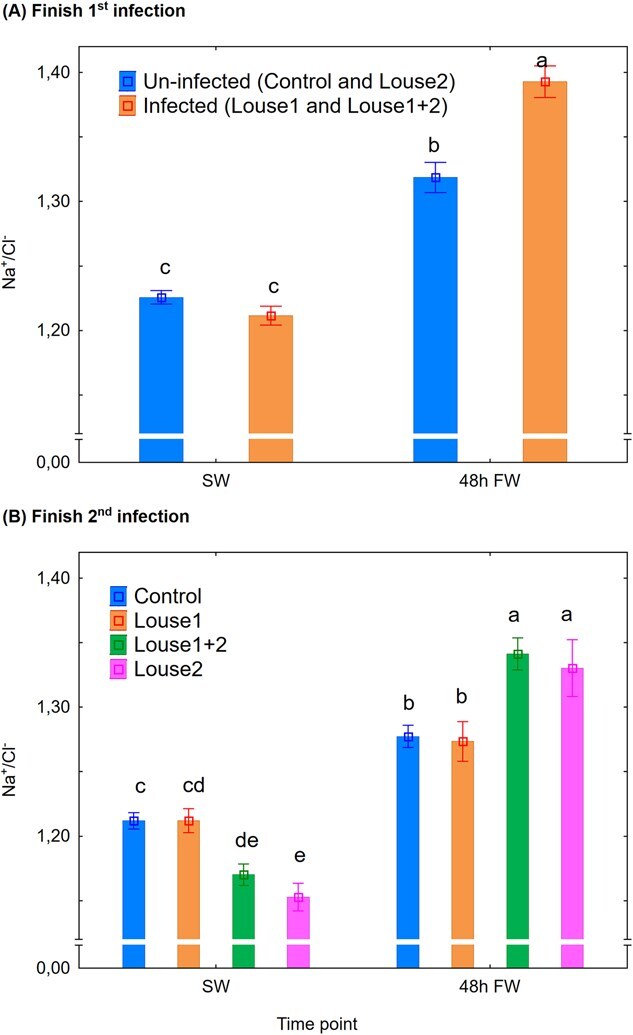
Plasma Na^+^/Cl^−^ ratio (mean ± SE). (**A**) Finish first infection. (**B**) Finish second infection. SW = at lice pre-adult stage in SW, 48 h FW = 48 h after change to FW. Different lowercase letters denote significant difference (*P* < 0.05) within each infection period.

Na^+^/Cl^−^ ratio: At Finish first infection, there were significant effects (mixed model ANOVA) of time point (*P* < 0.001) and the infection group × time point interaction (*P* < 0.001) on plasma Na^+^/Cl^−^ ratio, and both uninfected and infected fish had increased (Newman–Keuls *post hoc* test, *P* < 0.05) ratios by shift to FW when infected fish had a significantly higher ratio than uninfected fish (Fig. 8A). At Finish second infection, there were significant effects (mixed model ANOVA) of time point (*P* < 0.001) and the infection group × time point interaction (*P* < 0.01). At this stage, in SW, the Louse2 group had a significantly lower ratio than the control and Louse1 groups (Newman–Keuls *post hoc* test, *P* < 0.05) and the Louse1 + 2 had significantly lower ratio than the control group. Then all groups had significantly increased ratios by shift to FW, when the Louse1 + 2 and Louse2 groups had significantly higher ratios than the control and Louse1 groups (Fig. 8B).

### Linear regressions: Infection intensity vs. growth and plasma parameters

There were significant linear regressions for lice infection intensity vs. SGR, ΔCF, and all plasma parameters except glucose (data from infected fish from Finish first and second infection SW analysed together, Table 3); infection intensity had positive relationships with plasma pH, K^+^, Na^+^, Ca^2+^, Cl^−^ and osmolality, and negative relationships with SGR, ΔCF, and plasma lactate, SID and Na^+^/Cl^−^ ratio. Correlation graphs are shown in the Figs S7–S19.

**Table 3:** *r* values and *P*-values from simple linear regressions between the measured plasma parameters and lice infection intensity based on pooled data from infected fish sampled in SW at Finish first and second infection (*n* = 64)

Plasma parameter	*r* value	*P*-value
pH	0.6095	<0.0001
K^+^	0.4174	0.001
Ca^2+^	0.4274	<0.0001
Na^+^	0.6672	<0.0001
Cl^−^	0.7695	<0.0001
Osmolality	0.6762	<0.0001
Glucose	−0.1728	0.172
Lactate	−0.3708	0.003
SID	−0.5934	<0.0001
Na^+^/Cl^−^	−0.7074	<0.0001

### Lice infection did not affect GSI and sperm quality

Among maturing male trout, there was a tendency to lower GSI (mixed model ANOVA, *P* = 0.064) in lice-infected fish (5.0 ± 0.2%) compared to uninfected fish (5.9% ± 0.3%) at Finish first infection. At Finish second infection, however, GSI levels (2.4–2.6 ± 0.2–0.3%) of maturing males were equal (mixed model ANOVA, *P* = 0.973) between the four treatment groups.

When milt from the present mature males was used to fertilize eggs (not from this experiment), there were no significant effects of treatment on fertilization rate, or egg, fry or total mortalities (Table 4).

**Table 4:** Sperm quality estimates

	Group				*P*-value
Parameter	Control	Louse1	Louse1 + 2	Louse2	
Fertilization rate (%)	98 ± 1	98.2 ± 0.8	97.7 ± 1.3	99.5 ± 0.3	0.345
Mortality before hatch (%)	30.2 ± 6.4	26.8 ± 4.7	34 ± 8.1	22.7 ± 4.3	0.561
Mortality after hatch (%)	2.1 ± 1.4	0.9 ± 0.4	0.3 ± 0.3	0.7 ± 0.5	0.225
Mortality total (%)	31.8 ± 6.3	27.5 ± 4.5	34.2 ± 8.1	23.3 ± 4.3	0.550

Egg fertilization rate and embryo and fry mortality rates (mean ± SE) following fertilization with sperm from mature male uninfected (control) and lice-infected (Louse1, Louse2, Louse1 + 2) sea trout. The eggs used came from 3-year-old sea trout that did not belong to the present study.

## Discussion

The present study shows that lice infection and infection intensity have significant effects on the trout host’s growth rate, osmoregulation and acid–base regulation, but not semen quality. There was no strong evidence for possible impacts of lice reinfection after a period in FW.

### Repeated lice infection did not reduce lice infection success

Previous studies suggest that some brown trout populations may exhibit lower susceptibility and delayed development of salmon lice (Glover *et al.*, 2001, 2003). The developmental speed and infection success reported here were in accordance with reported numbers for Atlantic salmon and sea trout (Bjørn and Finstad, 1998; Hamre *et al.*, 2009, 2019). The experiment was, however, not specifically designed to investigate this. We did observe a non-significant but lower infection success in previously infected fish. In Atlantic salmon, reinfection has been reported to be affected by previous infections, but the effect appears to depend on experimental set-up and whether lice from the previous infection are still present (Ugelvik *et al.*, 2017, 2023). To our knowledge, reinfection in sea trout has not been previously tested, despite its ecological relevance. Future studies should expose naïve and previously infected trout in a common-garden setup within the same tanks to explore this question further. In the current study, there were no differences in mortality between infected and uninfected fish, although high-intensity salmon louse infections can cause mortality in sea trout (reviewed in Thorstad *et al.*, 2015).

### Lice infection reduced SGR

The current study showed that lice infection reduced SGR in sea trout, confirming the results from field studies using scale growth as a proxy for body growth (reviewed in Thorstad *et al.*, 2015). The effect of lice was only significant in female trout, but the magnitude of the effect was equal in male and female trout. All females were immature, whereas males were a mixture of immature and maturing individuals. Differential response based on maturity may have caused a larger variation in males. There were, however, no differences in body length and weight between the infected groups and the control group, which may be due to the initial large individual variation in body size in the studied population masking any smaller effect.

The reduction in SGR caused by lice infection was largest in the second infection round, which also had the highest infection intensity. It should however be noted that the fish had been in SW for 3 months before the start of the first infection growth period, while they had been in FW for 12 days before the start of the second infection growth period. This may have affected the growth rate of the fish. The control fish indeed had 2-fold higher SGR during the first compared to the second infection period. Nevertheless, the control group had 1.45-fold higher SGR than those infected with on average 0.29 lice g^−1^ during the first infection growth period, and 4.8- to 11.7-fold higher SGR than those infected with on average 0.61 lice g^−1^ during the second infection growth period. The effect on SGR was similar between fish that was infected for the first time and fish that had been previously infected during the second infection growth period. Compared to similar studies performed on other hatchery-reared salmonid postsmolts in the same facilities and with equal environmental conditions, uninfected Atlantic salmon had 2.3-fold higher SGR than those infected (mean intensity, 0.38 lice g^−1^) (Fjelldal *et al.*, 2020), while uninfected Arctic char (*Salvelinus alpinus*) had 16-fold higher SGR than those infected (mean intensity, 0.33 lice g^−1^) (Fjelldal *et al.*, 2019). Thus, the reported growth responses to salmon louse are similar in sea trout and Atlantic salmon and with much less negative effects than what was previously observed in char. There is a clear increase in the SGR response to infection as infection intensity increases from the first to the second infection in the presently studied sea trout, supported by a significant linear regression between infection intensity and SGR for both periods pooled (Fig. S7). Similar results have been shown in Atlantic salmon (Fjelldal *et al.*, 2020, 2022). Taken together, the studies demonstrate stronger growth retardation by lice in Arctic char compared to sea trout and Atlantic salmon, which is vital information for conservation.

When infections with salmon louse reduce the growth rate of wild sea trout, reduced fecundity may also be a consequence. Work on Atlantic salmon has suggested that lice infection can increase age at maturation caused by either increasing age at puberty or inducing higher mortality in early maturing fish (Vollset *et al.*, 2014). Indeed, modelling studies have indicated that the harmful effect of salmon louse on wild Atlantic salmon populations is primarily driven by elevated fish mortality, which can outweigh any potential ‘positive’ effects of elevated fecundity associated with increased age at maturation (Vollset and Krkosek, 2021). In the present study, lice infection had no detectable effect on fertilization rate or fry survival, even though mature male trout had been infected twice. The result suggests that lice infection does not impair sperm quality in sea trout after a period in FW. Whether sperm quality would be affected in heavily infected, fully mature males that are transferred to FW shortly before spawning remain unknown. In mature male chum salmon (*O. keta*), sodium concentration and osmolality are equal in blood and seminal plasmas and transfer from SW to FW reduces sodium, chloride and osmolality in both plasmas, but with a more pronounced effect in seminal plasma (Morisawa *et al.*, 1979).

### Lice infection elevated HSI

The current results showed that salmon louse infection increases HSI in sea trout, which is in agreement with an earlier study (Fjelldal *et al.*, 2023). In that study, a link to increased water content caused by elevated liver glycogen was suggested. Indeed, in Atlantic salmon, bacterial infection and development of coldwater vibriosis have been shown to reduce serum albumin, cholesterol and alkaline phosphatase, and increase HSI and liver water and lipid content, as well as plasma ALAT (Waagbø *et al.*, 1988). The liver is a main site for albumin production in fish, and infection with salmon louse copepodids decreases plasma albumin in sea trout (Grimnes and Jakobsen, 1996; Birkeland and Jakobsen, 1997; Dawson, 1998). Skugor *et al.* (2016) found lower plasma concentrations of cholesterol and several enzymes related to liver function, and hepatic expression of genes related to detoxification, metabolism and immune function in lice-infected compared to uninfected Atlantic salmon.

Taken together, these findings highlight the need for further research on the mechanism by which salmon lice affect the liver in salmonid hosts. Further studies should investigate how lice infection affects liver histology and gene expression/biological pathways in salmonids.

### Reduced hyper-osmoregulatory ability in lice-infected trout

Similar to the present findings, several studies have demonstrated reduced hypo-osmoregulatory ability in salmonid hosts in SW when lice reach the pre-adult stage (e.g. Birkeland and Jakobsen, 1997). However, the present study is the first to show an effect on hyper-osmoregulatory ability when lice-infected fish are transferred to FW. Reduced hyper-osmoregulatory ability has been associated with elevated mortality rate in Atlantic salmon (Imsland *et al.*, 2011) and may help explain the mortality observed in lice-infected wild sea trout returning to FW (Birkeland, 1996). Imsland *et al.* (2011) reported a mean plasma Cl^−^ level of 125 mmol l^−1^ in Atlantic salmon, which had reduced hyper-osmoregulatory ability and raised mortality. Equal levels have been reported in sea trout 48 h after a direct switch from SW to FW (Fjelldal *et al.*, 2023). In the present study, however, mean plasma Cl^−^ levels after 48 h in FW were 118 and 109 mmol l^−1^ in control and infected trout, respectively, after the first infection, and 121, 121, 114 and 110 mmol l^−1^ in the control, Louse1, Louse2 and Louse1 + 2 groups, respectively, after the second infection. These findings indicate that sea trout already face challenges with hyper-osmoregulation following a direct transfer from SW to FW, and salmon lice can further exacerbate this impairment. In the present facility, the FW was supplemented with UV-treated SW to increase buffer capacity, resulting in a salinity of ~ 0.8 ppt. In contrast, the natural river water from which the fish stock originates from is ion-poor. This difference may have masked potential FW-related mortality effects in the current study. Further studies should therefore examine how water salinity and temperature influence osmoregulation in sea trout infected with pre-adult or adult lice. Such knowledge is vital for conservation efforts and the understanding whether and under what conditions wild sea trout can withstand heavy lice burdens.

Reduced hyper-osmoregulatory ability in FW may be linked to lice-associated upregulation of hypo-osmoregulatory capacity in the fish. Elevated gill NKA enzyme activity (Nolan *et al.*, 1999) and chloride cell proliferation (Farrell, 2011) have been reported in Atlantic salmon infected with salmon louse. Thus, lice infection can elevate the host’s hypo-osmoregulatory capacity, which in theory can impede their hyper-osmoregulatory ability if transferred to FW. Indeed, when Atlantic salmon smoltifies, their development of hypo-osmoregulatory ability may be associated with a disturbance in hyper-osmoregulatory mechanisms shown by reduced plasma Cl^−^ levels in FW at peak smoltification (Arnesen *et al.*, 2003). Such effects of lice will most probably disappear when lice die following exposure to FW. The effect of lice on the Louse1 group was indeed transient as this group had equal hyper-osmoregulatory ability as the control group after the second infection.

### Lice infection affects acid–base regulation

The current study is the first to show an effect of salmon louse infection on host acid–base regulation. Lice infection elevated plasma pH and SID in SW and also increased plasma pH after 48 h in FW. The result may suggest that the overall effect of lice infection on acid–base regulation was stronger in SW than after 48 h in FW, which is not unexpected given the substantial loss of lice observed at that time point (personal observation).

The differing responses of plasma SID compared to pH to lice raises the question whether SID is a reliable indicator for acid–base disturbances in fish, particularly in FW. However, Maxime *et al.* (1990) found a similar time course of the changes in blood pH and SID in Atlantic salmon transferred from SW to FW. Based on this and that plasma lactate did not change with transfer to FW, the authors suggested a disequilibrium in extracellular Na^+^, K^+^ and Cl^−^ as an underlying factor. In contrast, in the present study, lice-infected but not uninfected fish showed elevated plasma lactate after 48 h in FW, which may have contributed to the contrasting results between the studies. In fish, the gill is the main organ for osmoregulation and acid–base regulation (reviewed in Yan and Hwang, 2019). Indeed, the current study shows reduced hypo-osmoregulatory and hyper-osmoregulatory ability by lice in SW and FW, respectively, alongside elevated plasma pH by lice both in SW and FW. Thus, the gill must be considered as a key organ in the mechanisms behind the hosts pathophysiological responses to lice.

Plasma pH increases on transfer from SW to FW (current study; Maxime *et al.*, 1990, 1991). The mechanism behind is linked to the tight connection between acid–base regulation and osmoregulation in the fish gill (Krogh, 1939; Wilkes and McMahon, 1986), where Na^+^ and Cl^−^ are exchanged with H^+^ and HCO_3_^−^ to maintain an electroneutral charge movement across the gill epithelia (Zimmer and Perry, 2022). Also, the current plasma Na^+^/Cl^−^ ratio increased on change from SW to FW in both lice-infected and uninfected sea trout, and is similar to previously reported observations in rainbow trout (Maxime *et al.*, 1990). Moreover, compared to uninfected control, lice infection reduced and increased plasma Na^+^/Cl^−^ ratio in SW and FW, respectively, caused by a stronger effect of lice infection on Cl^−^ than on Na^+^ concentration. However, a rationale connecting this evidence to the observed elevated plasma pH by lice is currently difficult to construct and should be explored in further studies.

## Conclusion

In sea trout, salmon louse infection can reduce growth rate, increase HSI, elevate plasma pH and disturb acid base regulation, and reduce hypo-osmoregulatory and hyper-osmoregulatory abilities in SW and FW, respectively. Lice infection has a stronger effect on the host’s Cl^−^ compared to Na^+^ regulation. Lice reinfection after a period in FW did not impact on these results. Future studies should examine how water salinity and temperature affect these endpoints in lice-infected sea trout and go more in depth on histological and molecular hepatic changes following lice infection. To evaluate the transferability of the present laboratory results to nature and their applicability for management and conservation, it should also be explored if and how the level of lice impacts acid–base regulation and osmoregulation in wild sea trout that have recently entered FW in a river habitat.

## Supplementary Material

Web_Material_coaf028

## Data Availability

Data will be made available on request.
