# Supplementary material for: Physiological responses in sea trout to repeated salmon louse infections and freshwater
Source: Conserv Physiol. 2025 Nov 25;13(1):coaf080. doi: 10.1093/conphys/coaf080 (PMC12649749; doi:10.1093/conphys/coaf080)
Supplement: Web_Material_coaf028 [file web_material_coaf028.pdf]

Supplementray Material

Sup. Fig. 1

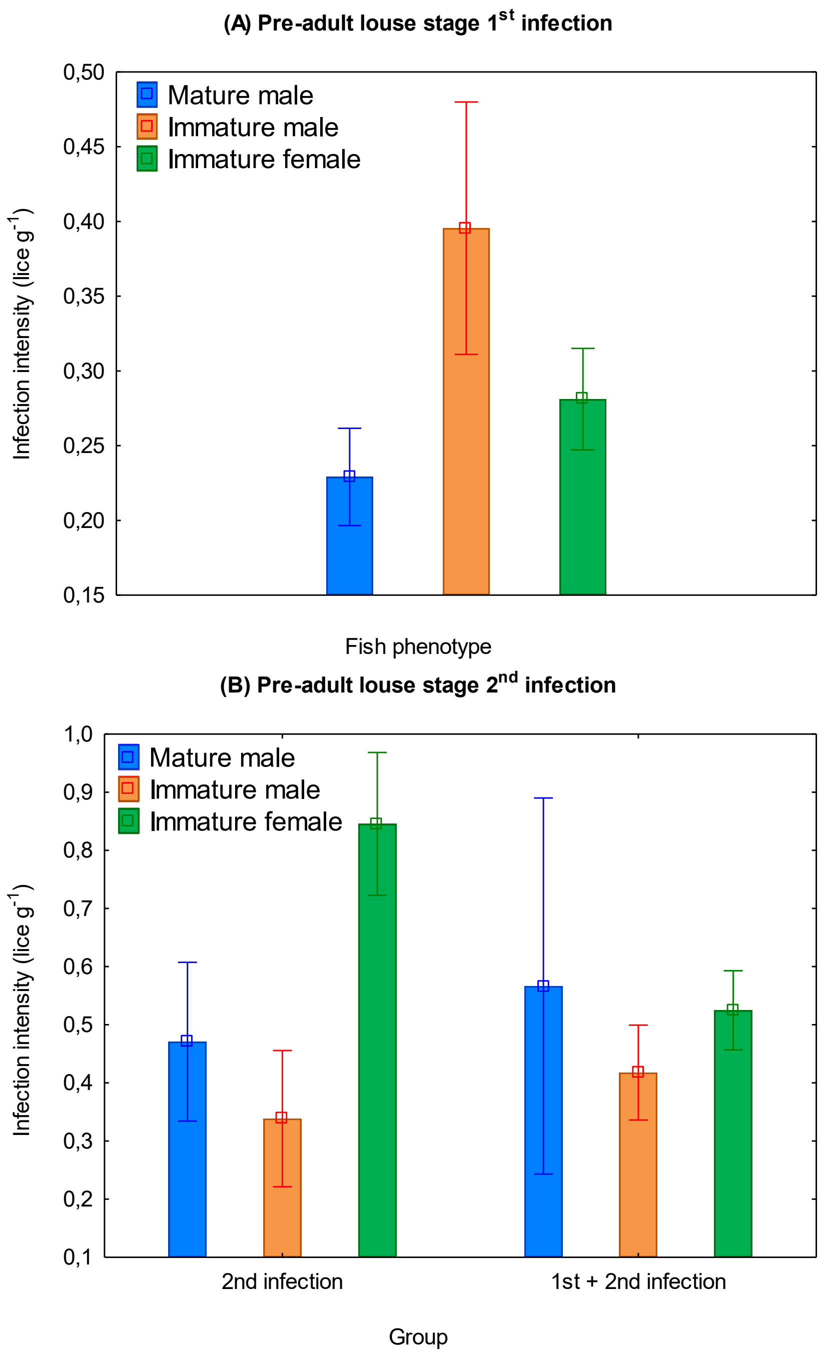

Sup. Fig. 1. Infection intensity (lice g^{-1}, mean ± SE). (A) Finish 1st infection before change from SW to FW. (B) Finish 2nd infection before change from SW to FW.

Sup. Fig. 2

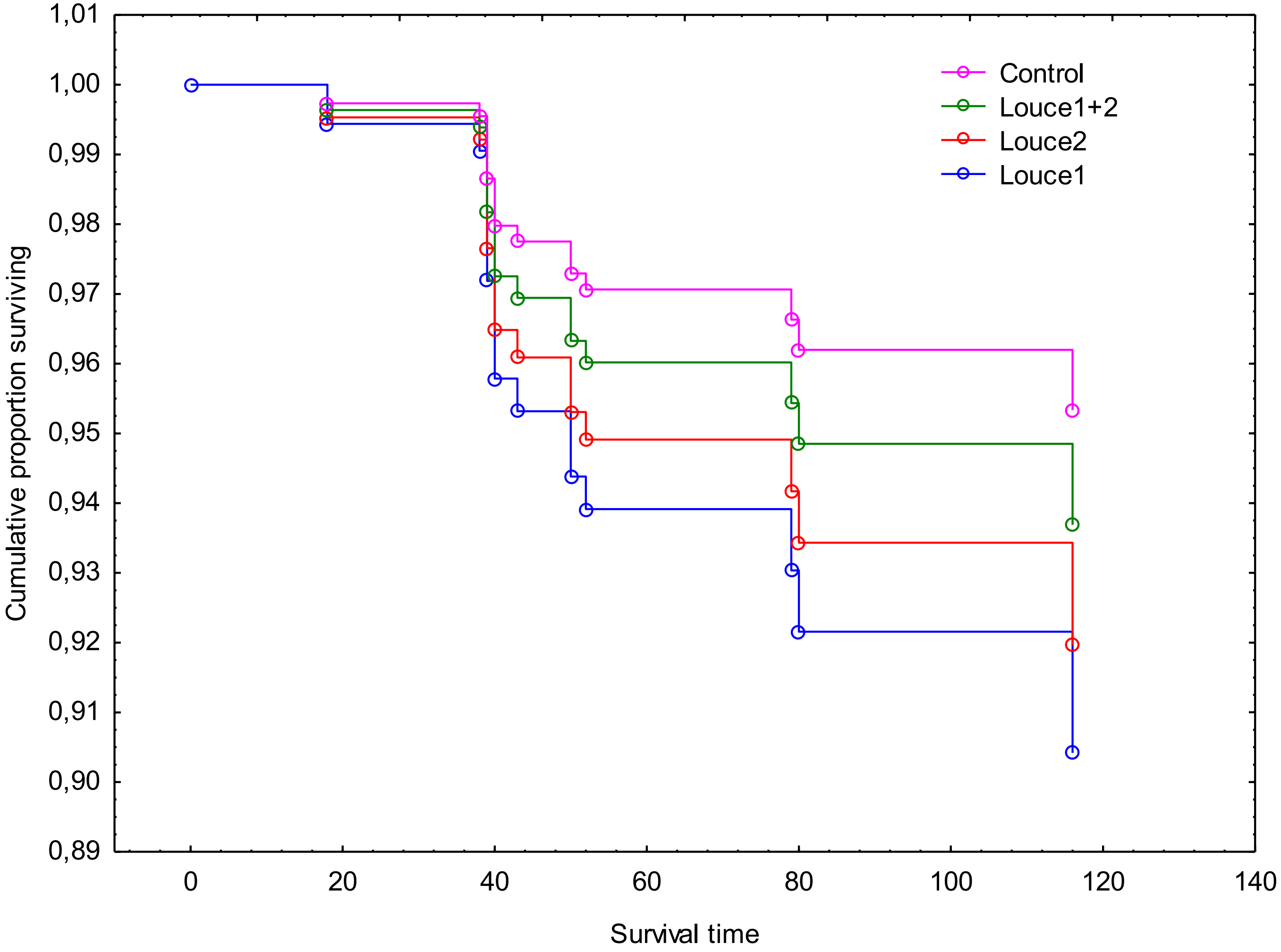

Sup. Fig. 2. Survival plot for un-infected control (Control) and salmon louse infected sea trout. The trout were infected using three different infection regimes: Infected during 1st infection (Louse1), infected during 2nd infection (Louse2), or infected during both the 1st and 2nd infection (Louse1+2).

Sup. Fig. 3

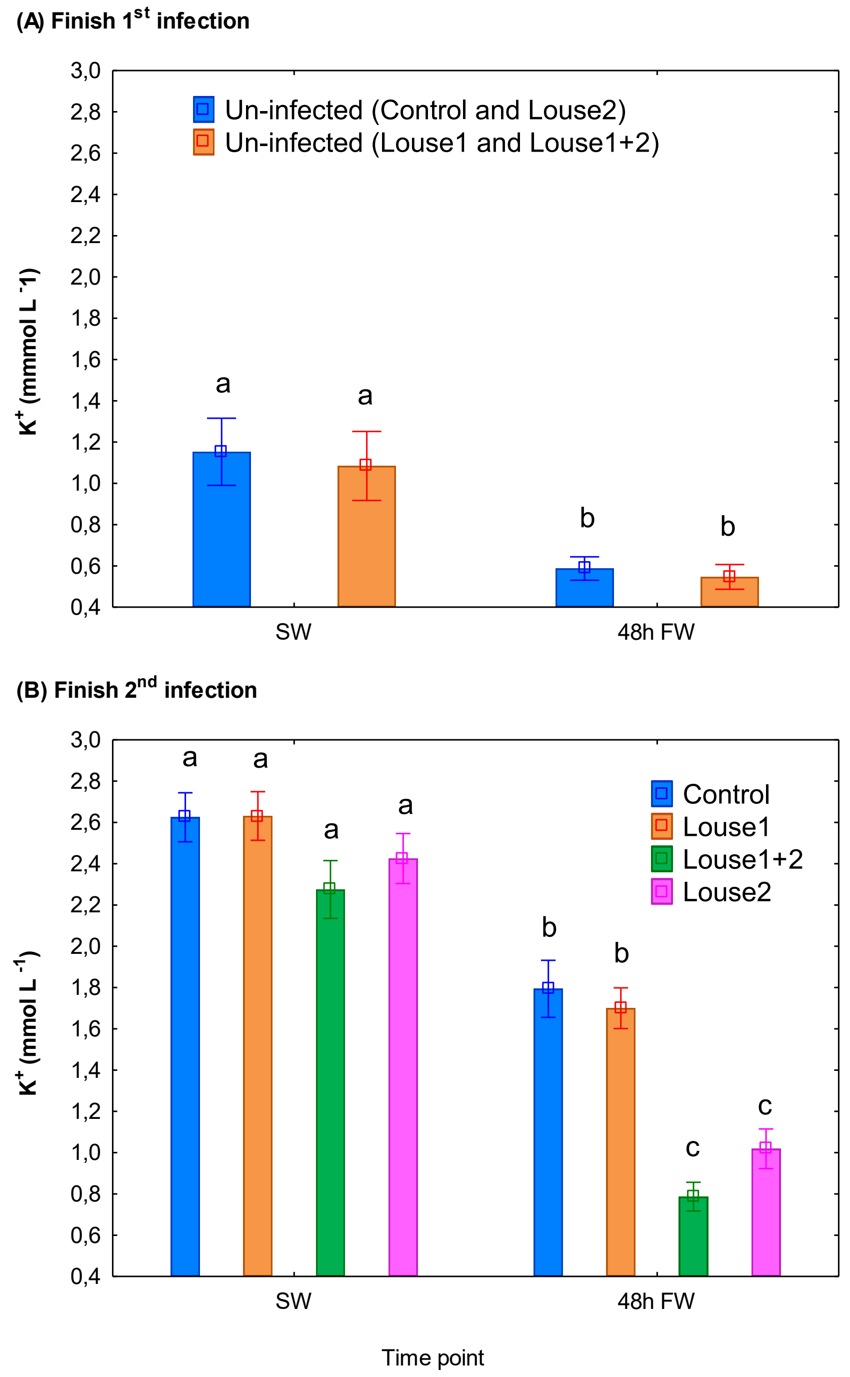

Sup. Fig. 3. Plasma K^+ (mean ± SE). (A) Finish 1st infection. (B) Finish 2nd infection. SW = at lice pre-adult stage, 48h FW = 48h after change to FW.

Sup. Fig. 4

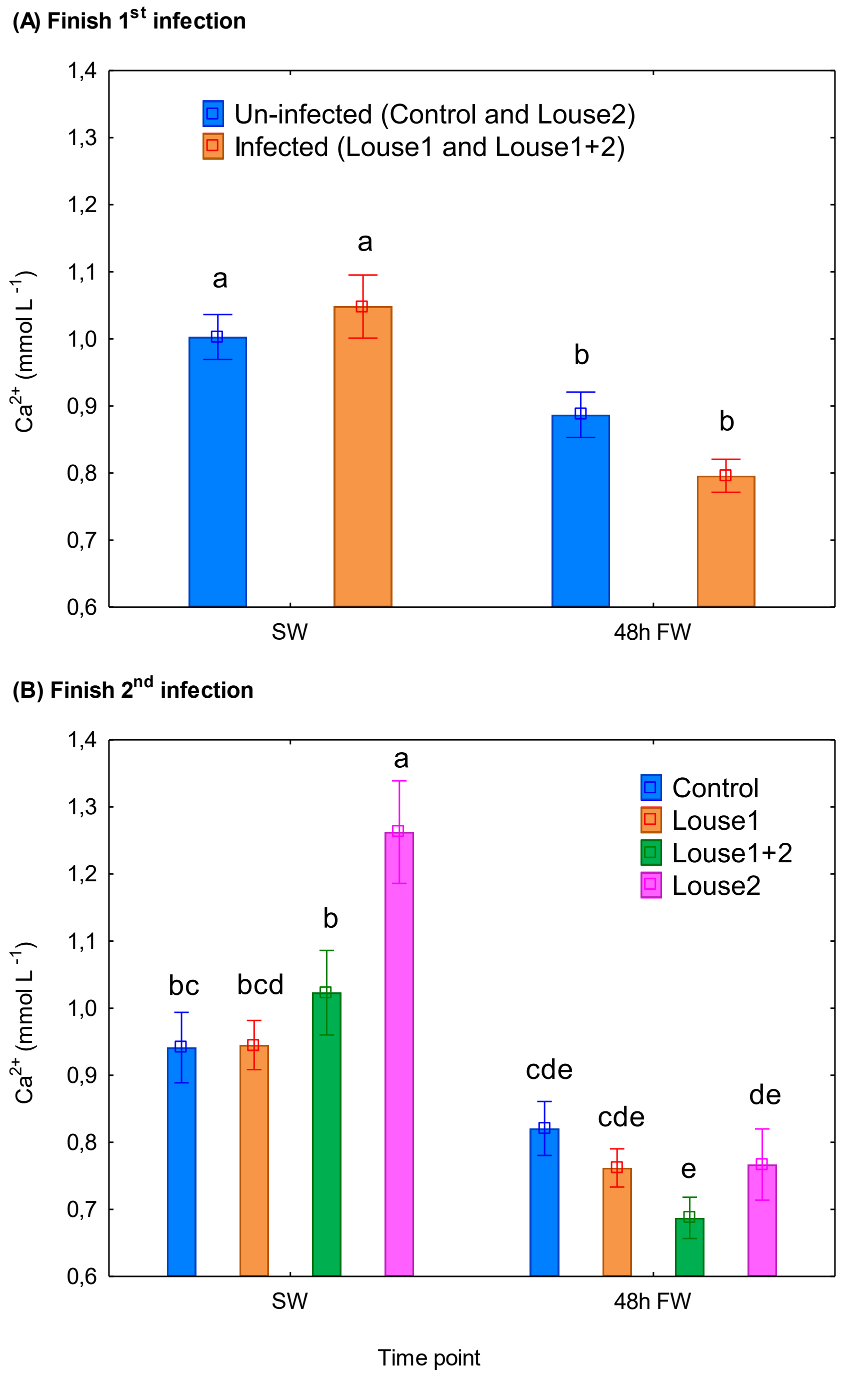

Sup. Fig. 4. Plasma Ca^{2+} (mean ± SE). (A) Finish 1st infection. (B) Finish 2nd infection. SW = at lice pre-adult stage, 48h FW = 48h after change to FW.

Sup. Fig. 5

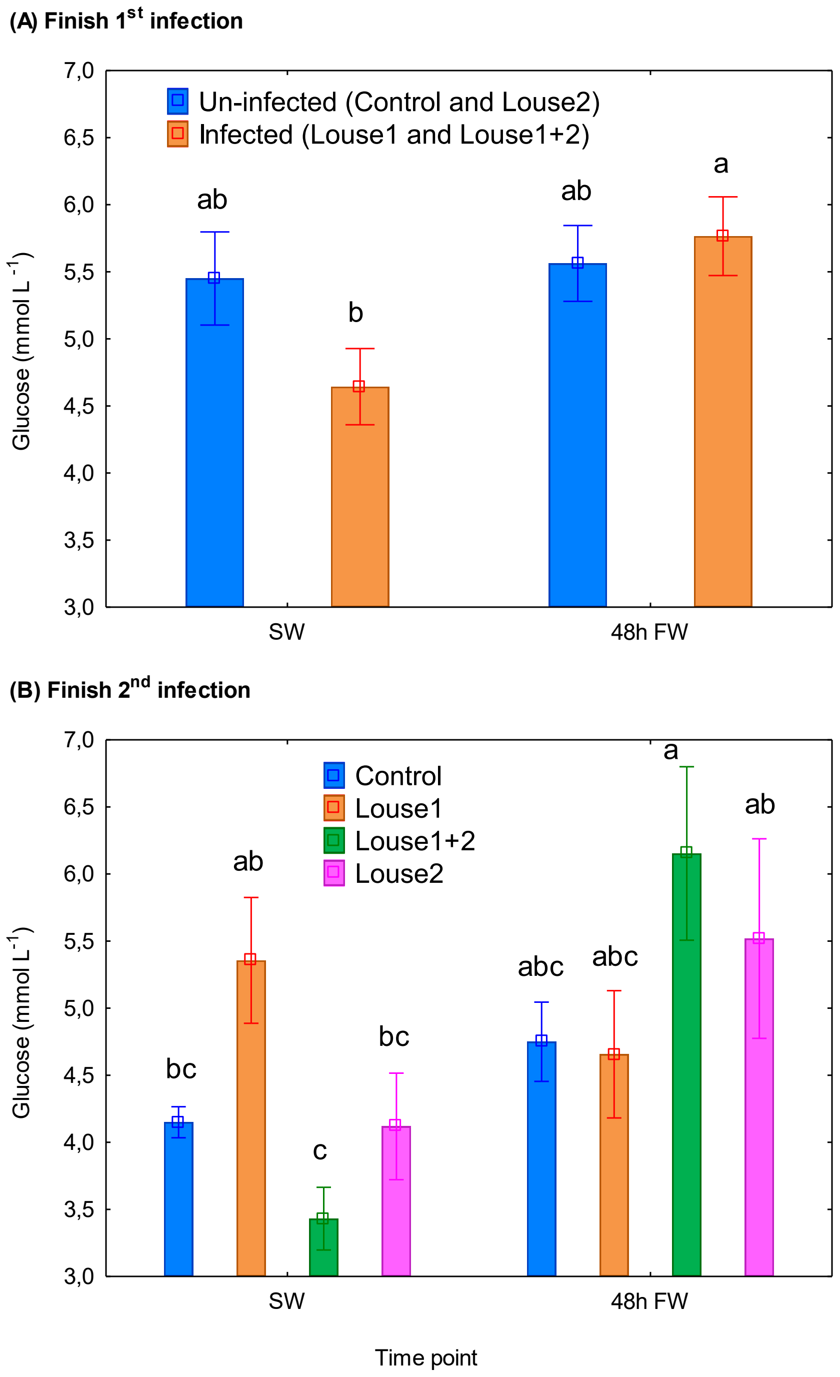

Sup. Fig. 5. Plasma glucose (mean ± SE). (A) Finish 1st infection. (B) Finish 2nd infection. SW = at lice pre-adult stage, 48h FW = 48h after change to FW.

Sup. Fig. 6

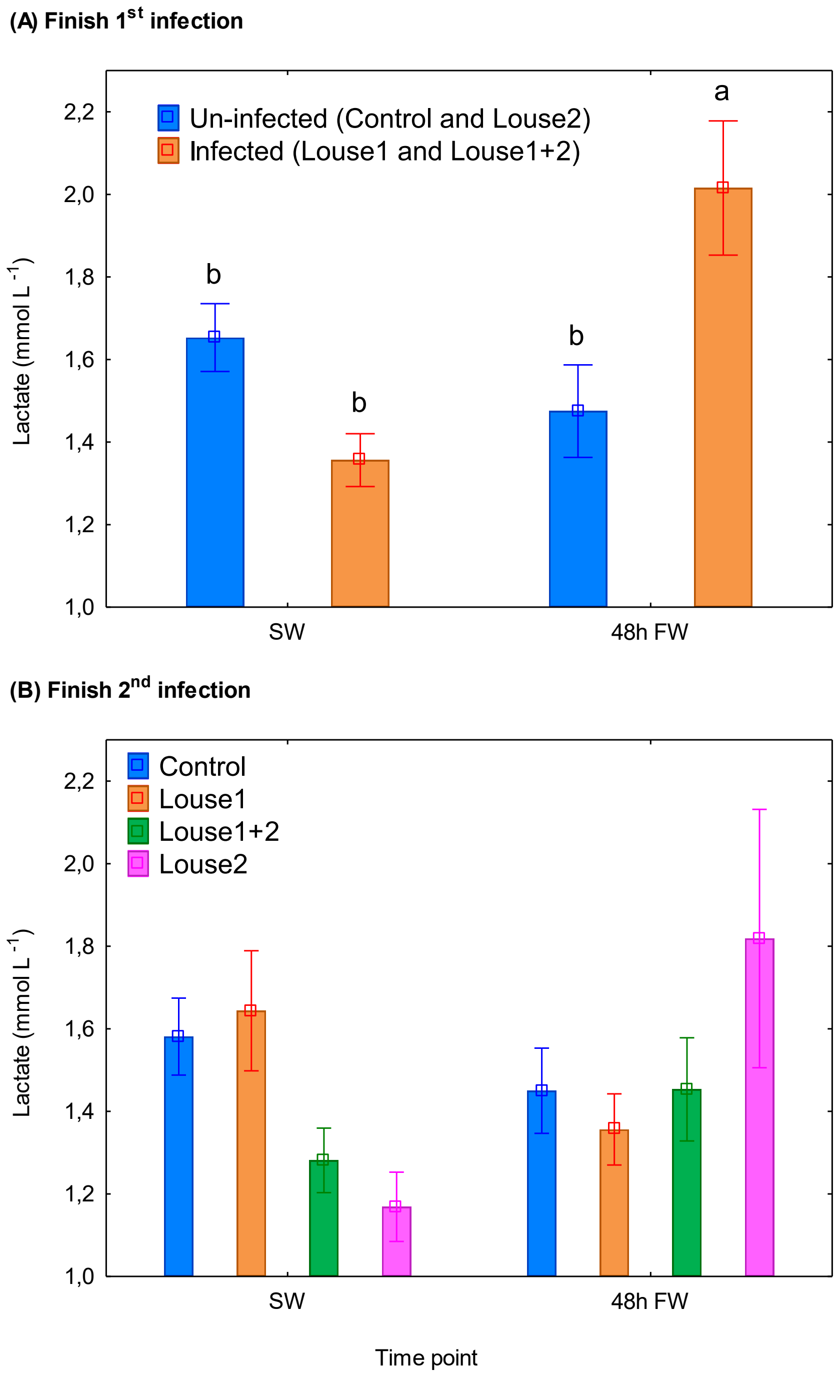

Sup. Fig. 6. Plasma lactate (mean ± SE). (A) Finish 1st infection. (B) Finish 2nd infection. SW = at lice pre-adult stage, 48h FW = 48h after change to FW.

Sup. Fig. 7

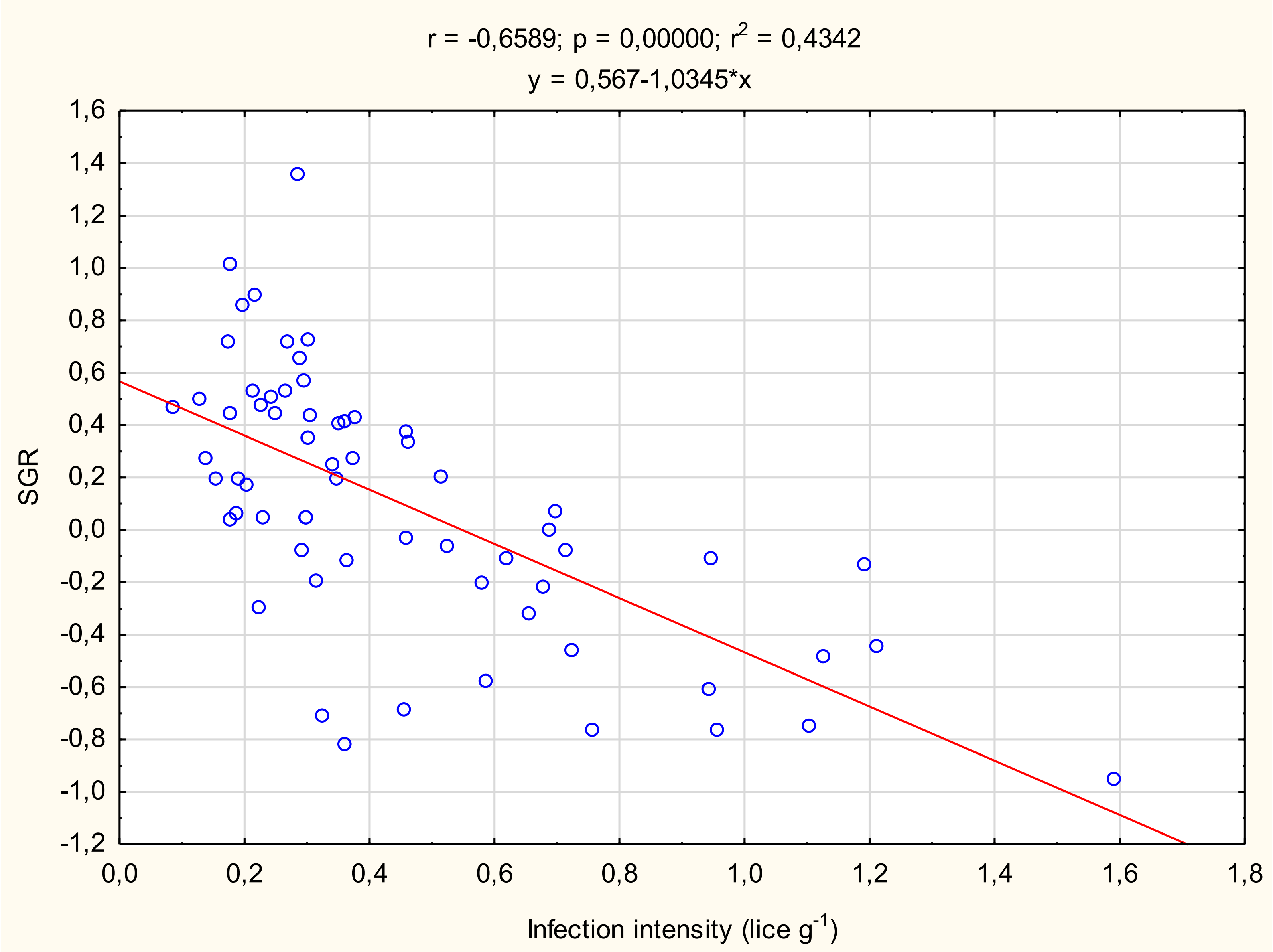

Sup. Fig. 7. Simple linear regression between lice infection intensity (lice g-1) and SGR (in the period from infection to the lice pre-adult stage).

Sup. Fig. 8

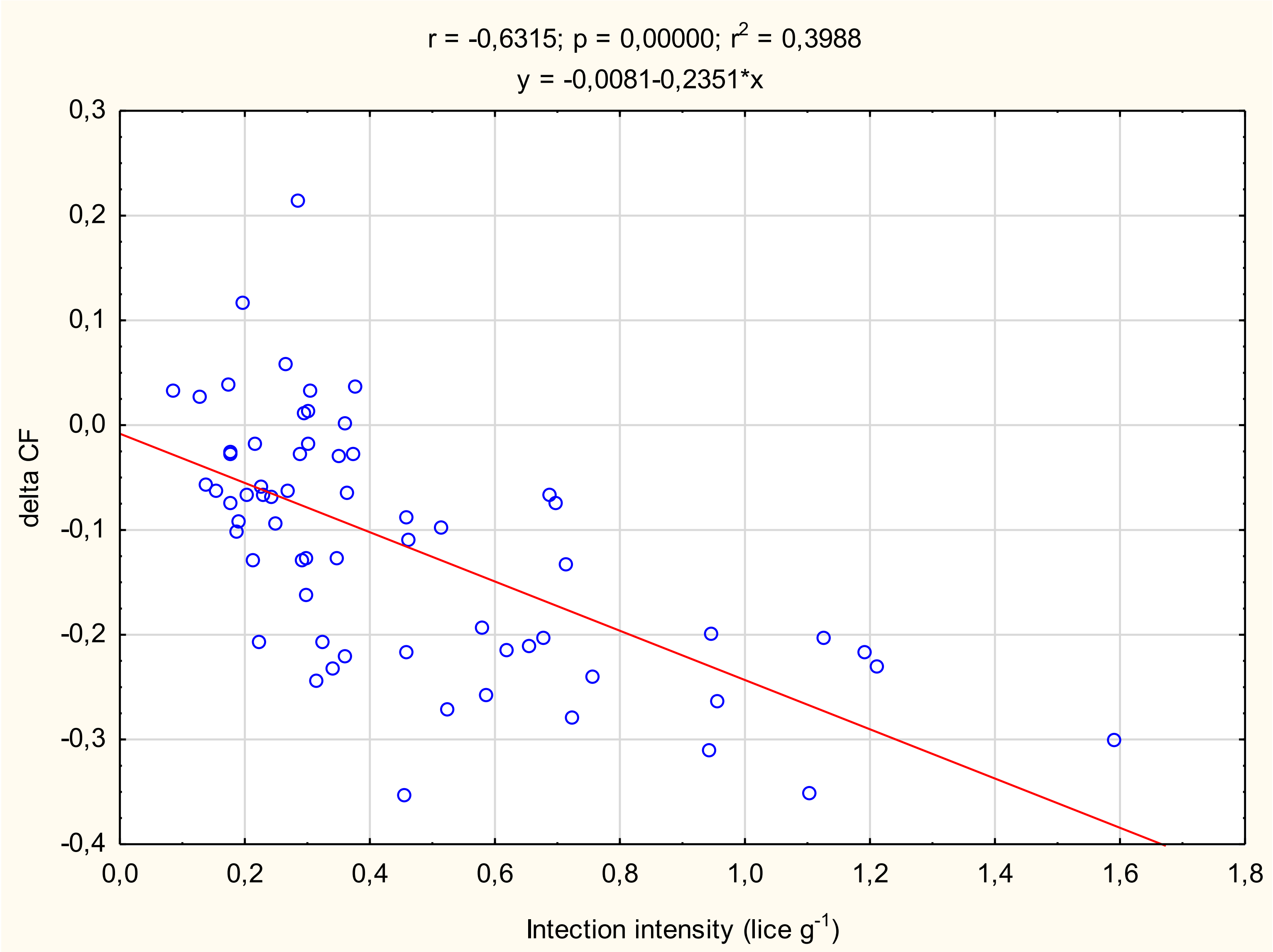

Sup. Fig. 8. Simple linear regression between lice infection intensity (lice g-1) and ΔCF (in the period from infection to the lice pre-adult stage).

Sup. Fig. 9

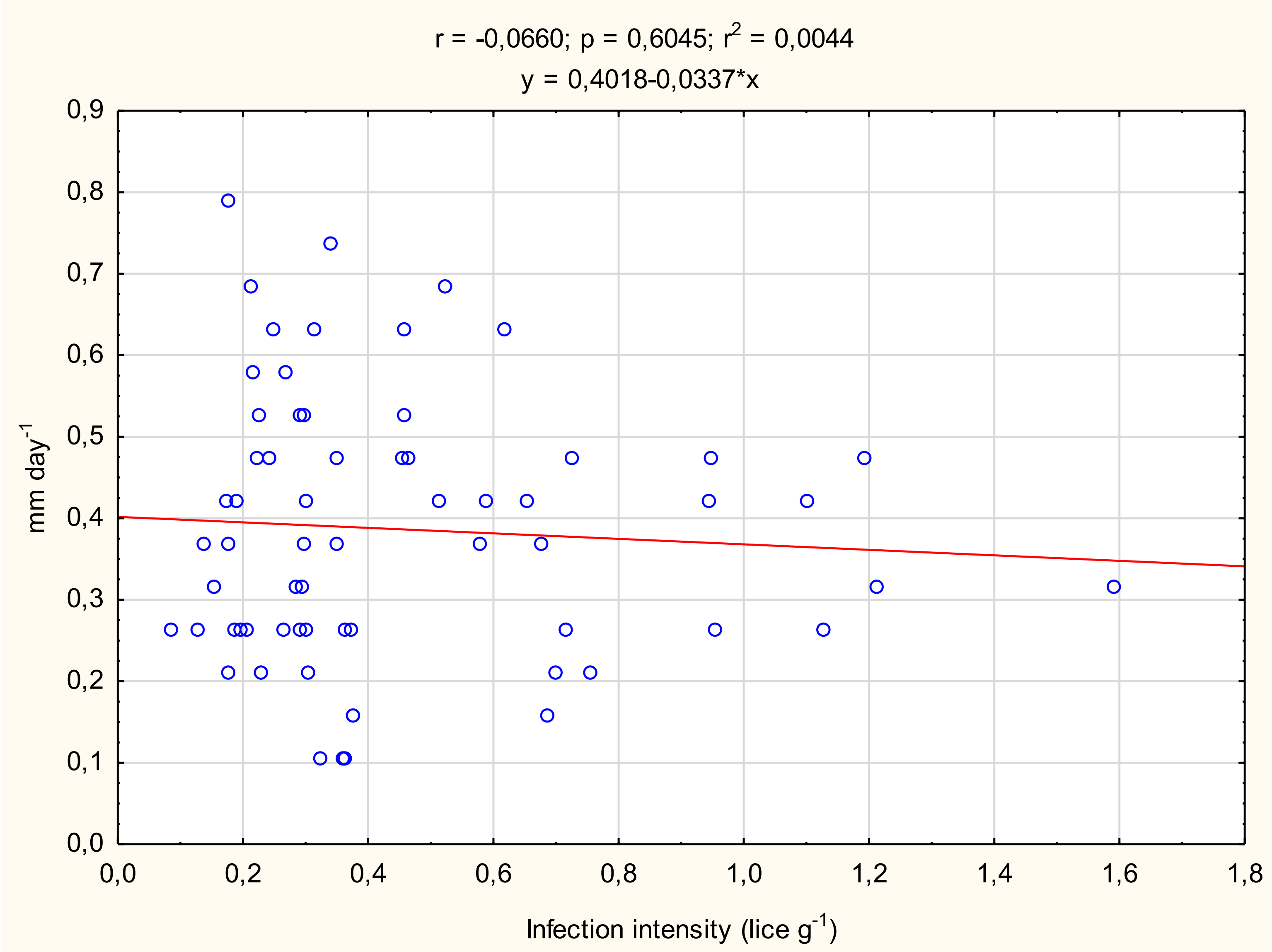

Sup. Fig. 9. Simple linear regression between lice infection intensity (lice g-1) and mm day-1 (in the period from infection to the lice pre-adult stage).

Sup. Fig. 10

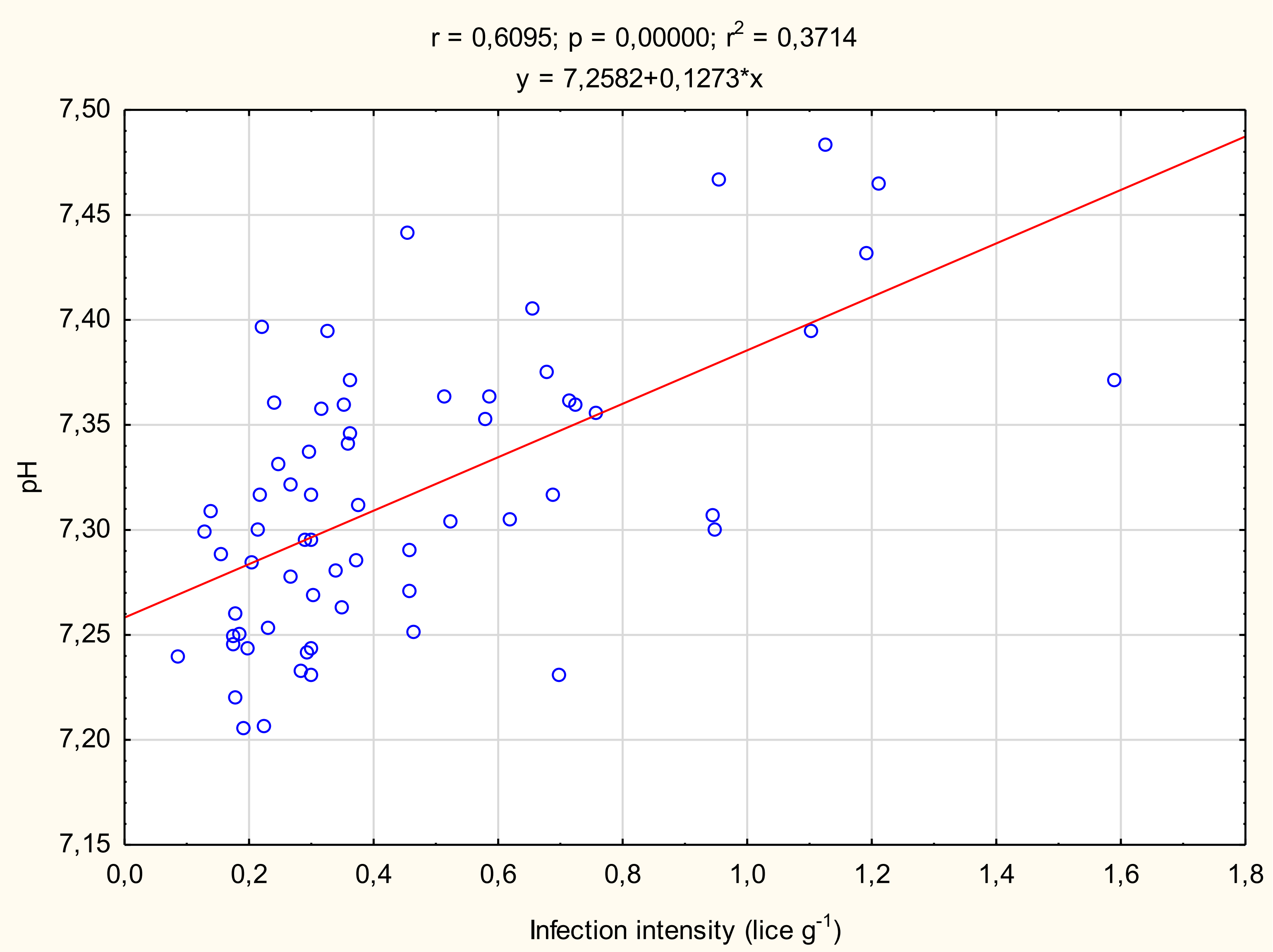

Sup. Fig. 10. Simple linear regression between lice infection intensity (lice g-1) and plasma pH.

Sup. Fig. 11

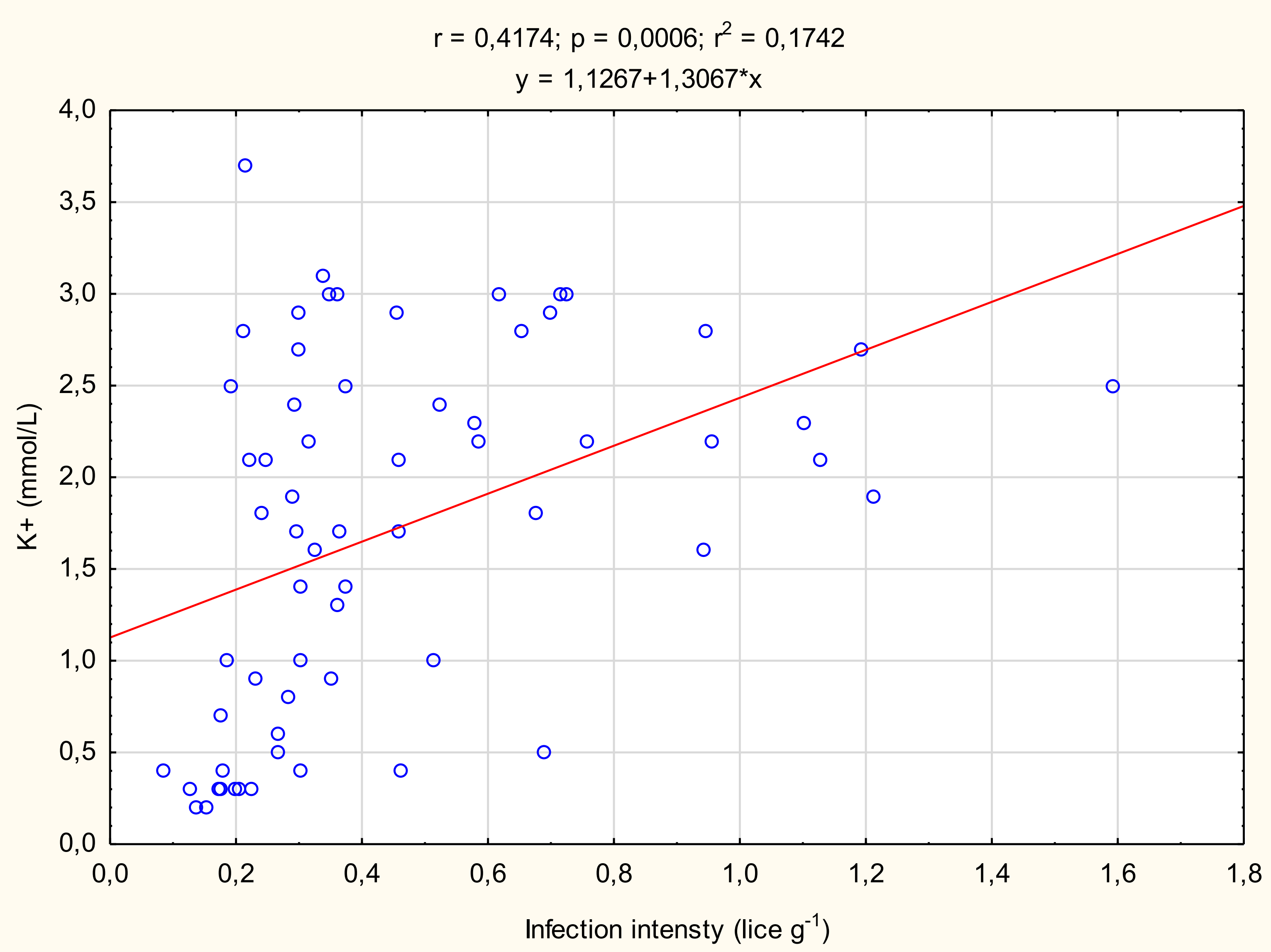

Sup. Fig. 11. Simple linear regression between lice infection intensity (lice g-1) and plasma K+.

Sup. Fig. 12

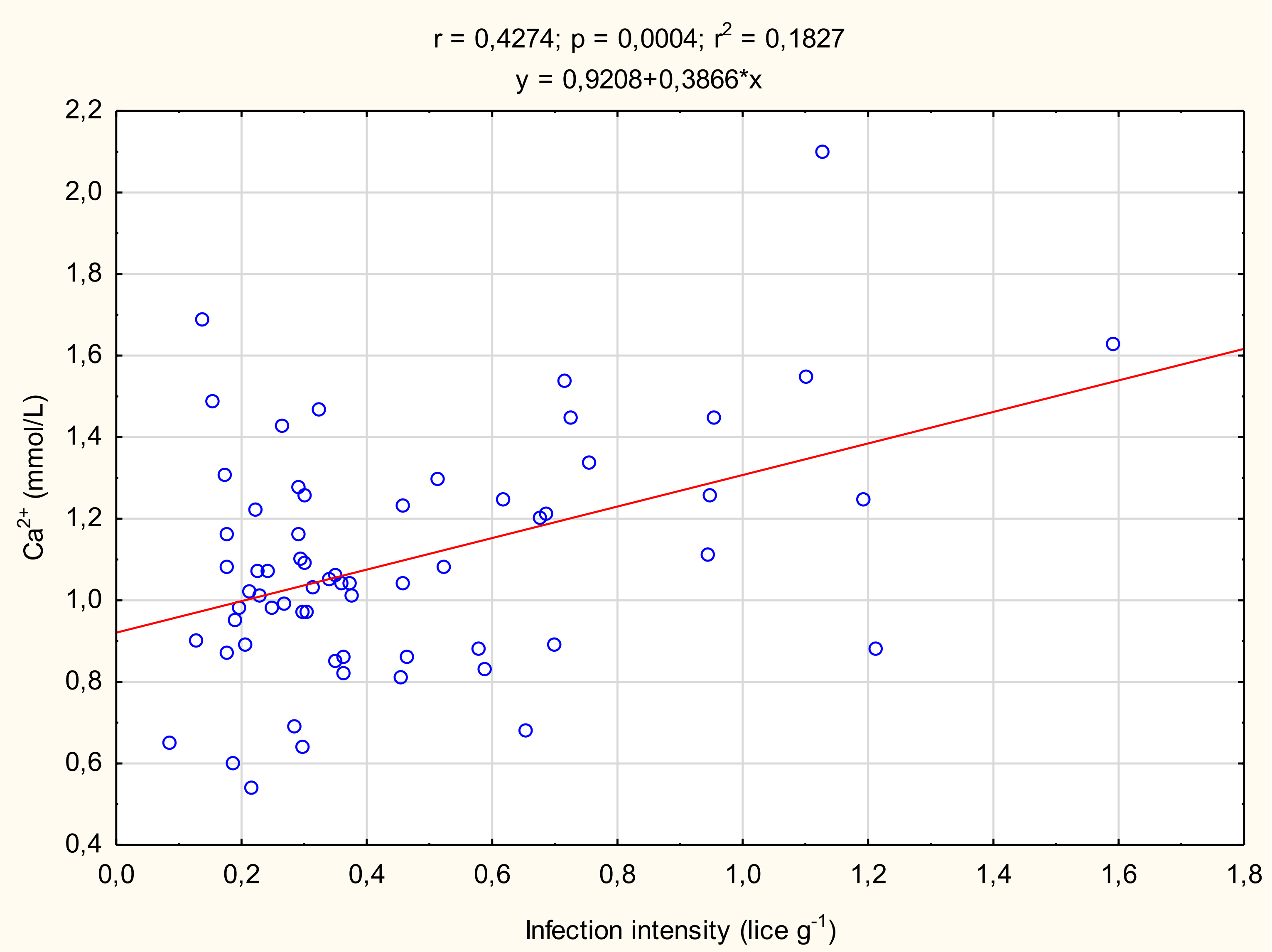

Sup. Fig. 12. Simple linear regression between lice infection intensity (lice g-1) and plasma Ca2+.

Sup. Fig. 13

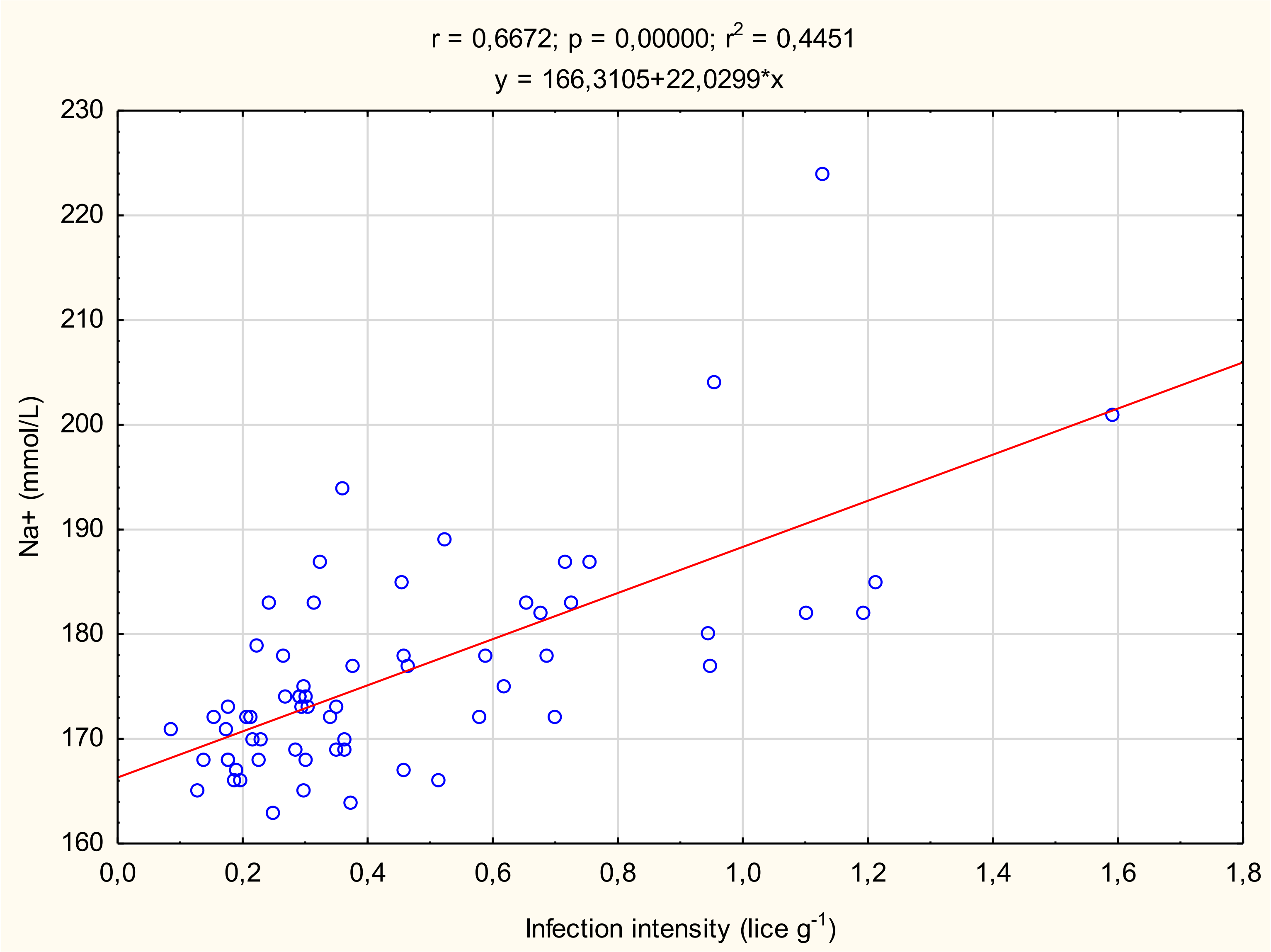

Sup. Fig. 13. Simple linear regression between lice infection intensity (lice g-1) and plasma Na+.

Sup. Fig. 14

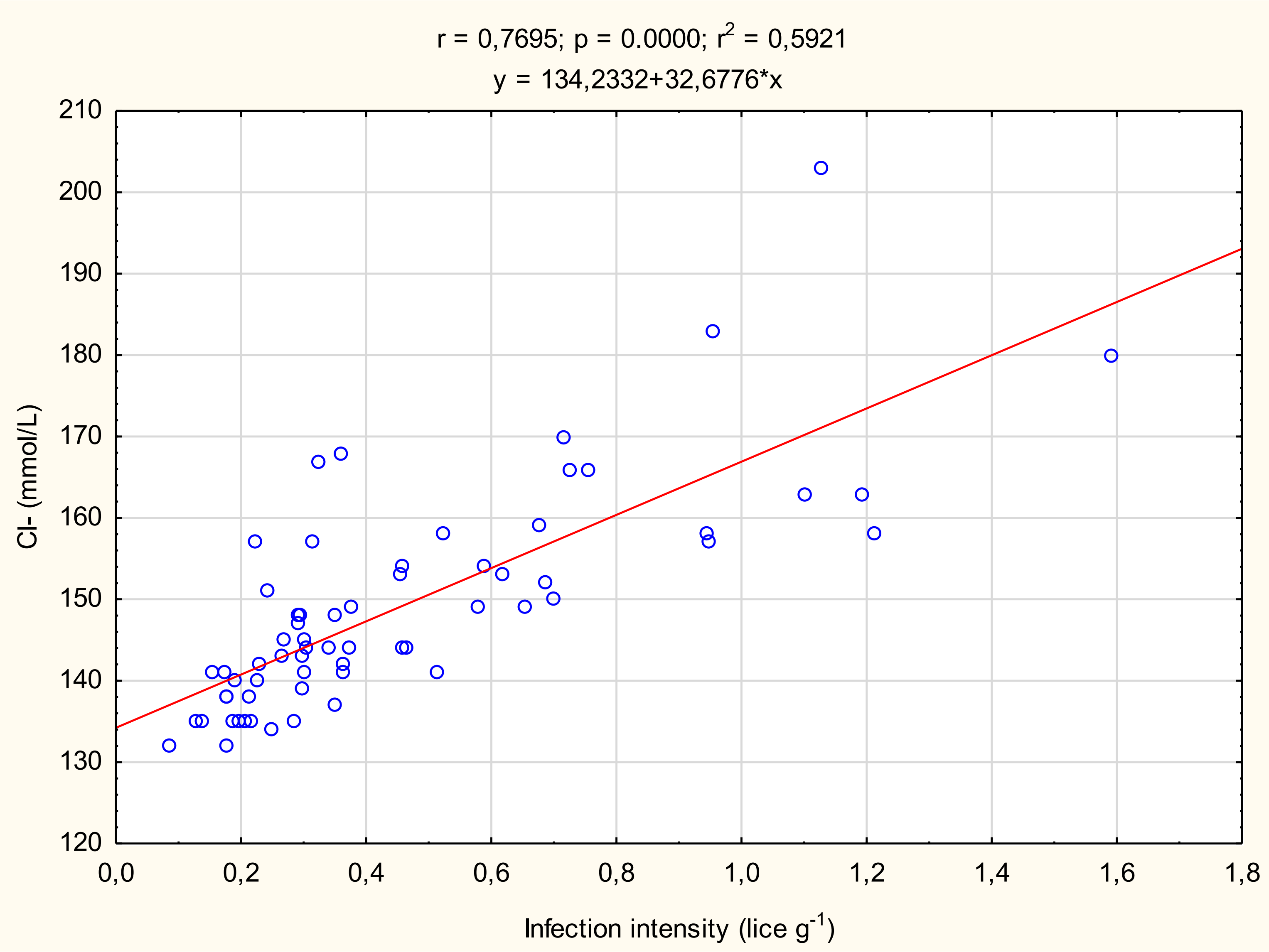

Sup. Fig. 14. Simple linear regression between lice infection intensity (lice g-1) and plasma Cl-.

Sup. Fig. 15

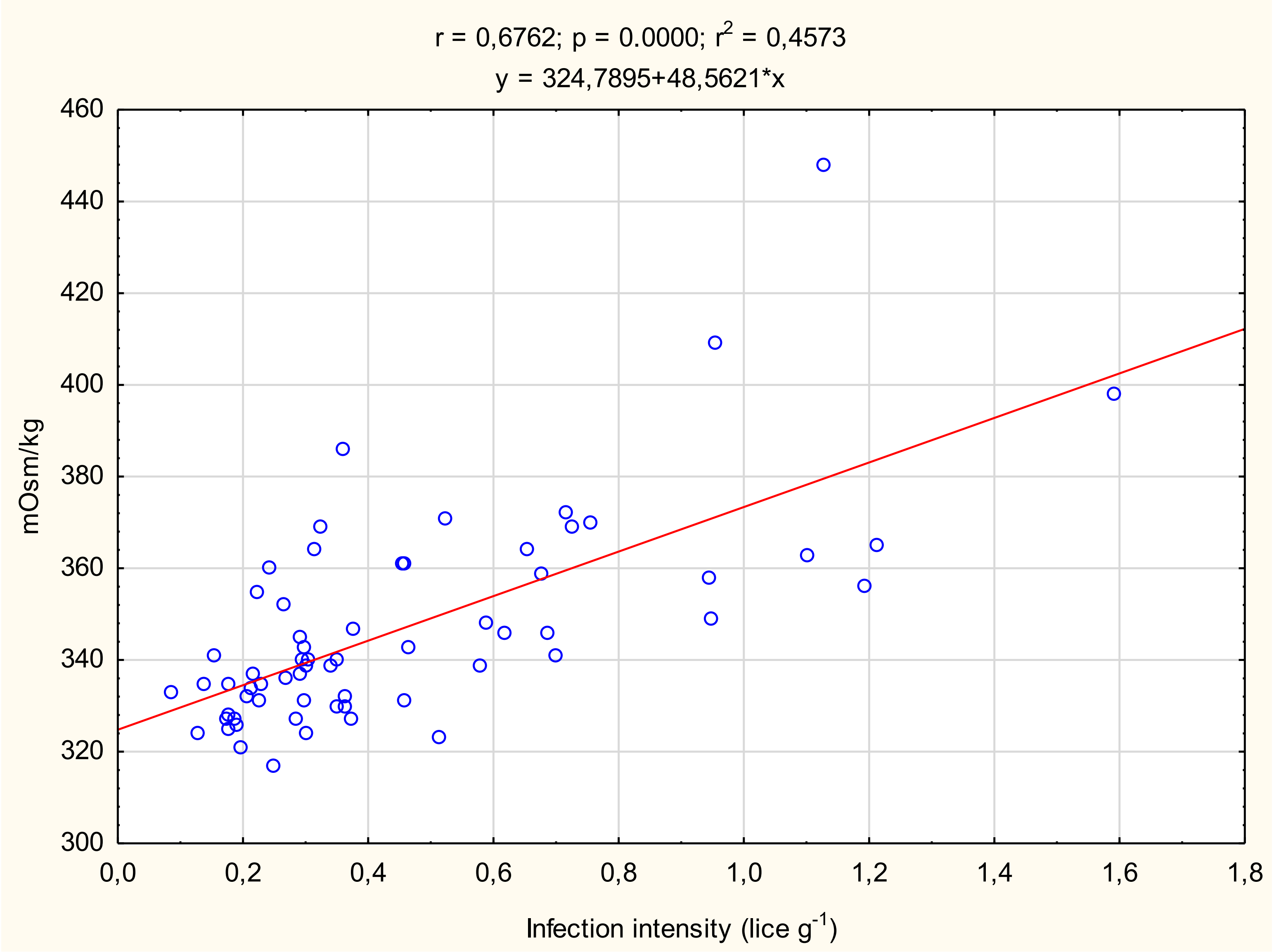

Sup. Fig. 15. Simple linear regression between lice infection intensity (lice g-1) and plasma osmolality.

Sup. Fig. 16

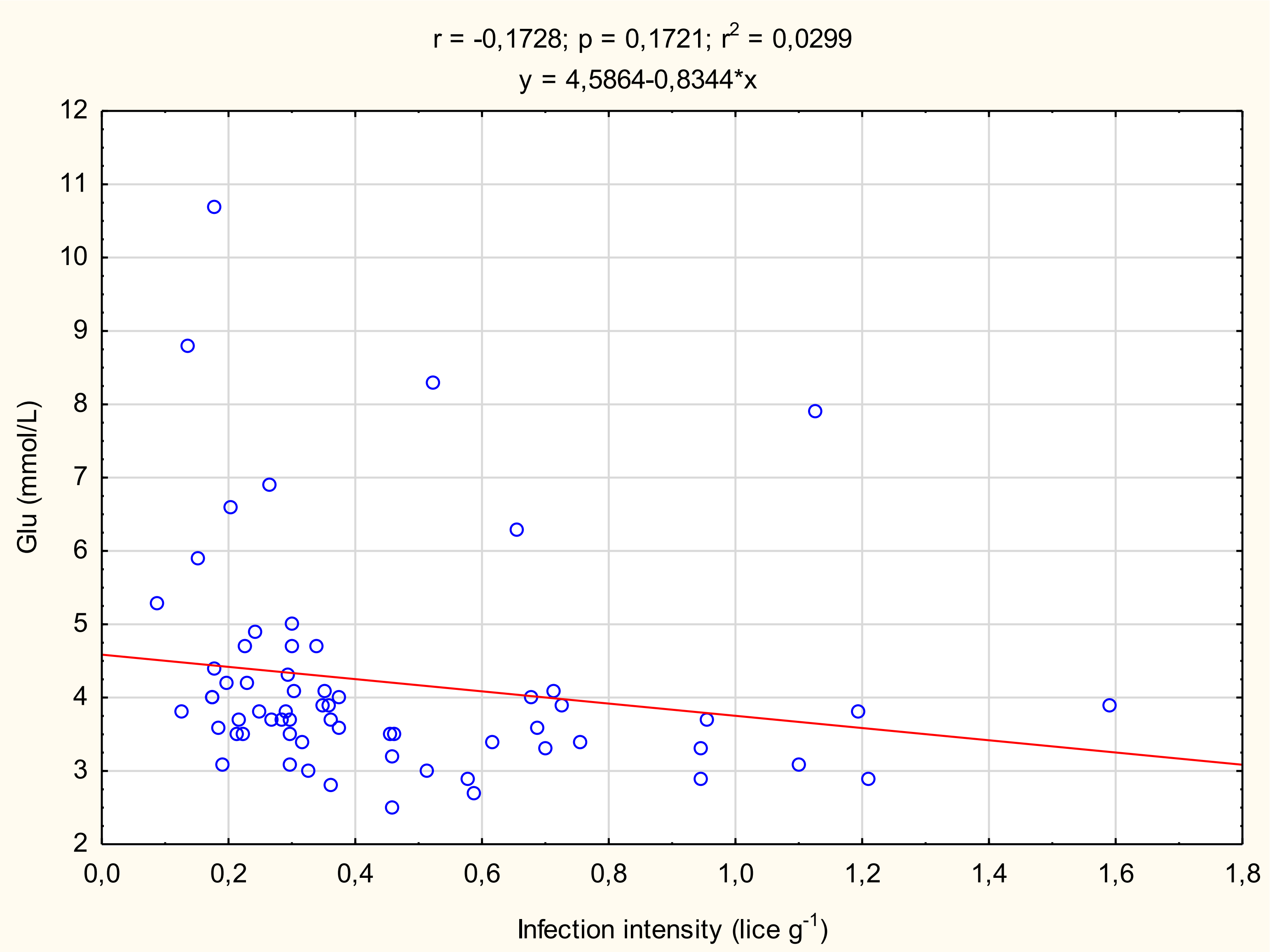

Sup. Fig. 16. Simple linear regression between lice infection intensity (lice g-1) and plasma glucose.

Sup. Fig. 17

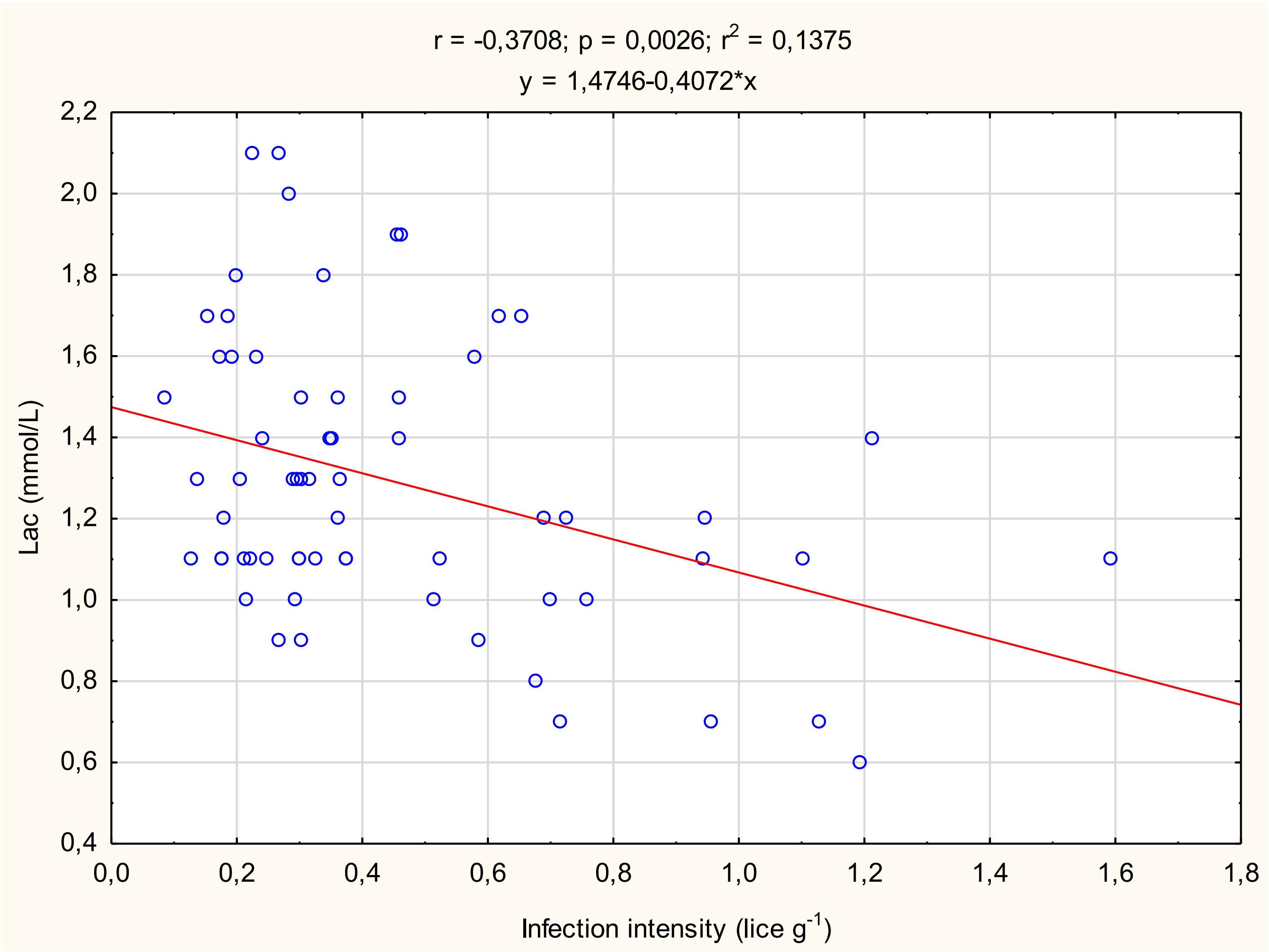

Sup. Fig. 17. Simple linear regression between lice infection intensity (lice g-1) and plasma lactate.

Sup. Fig. 18

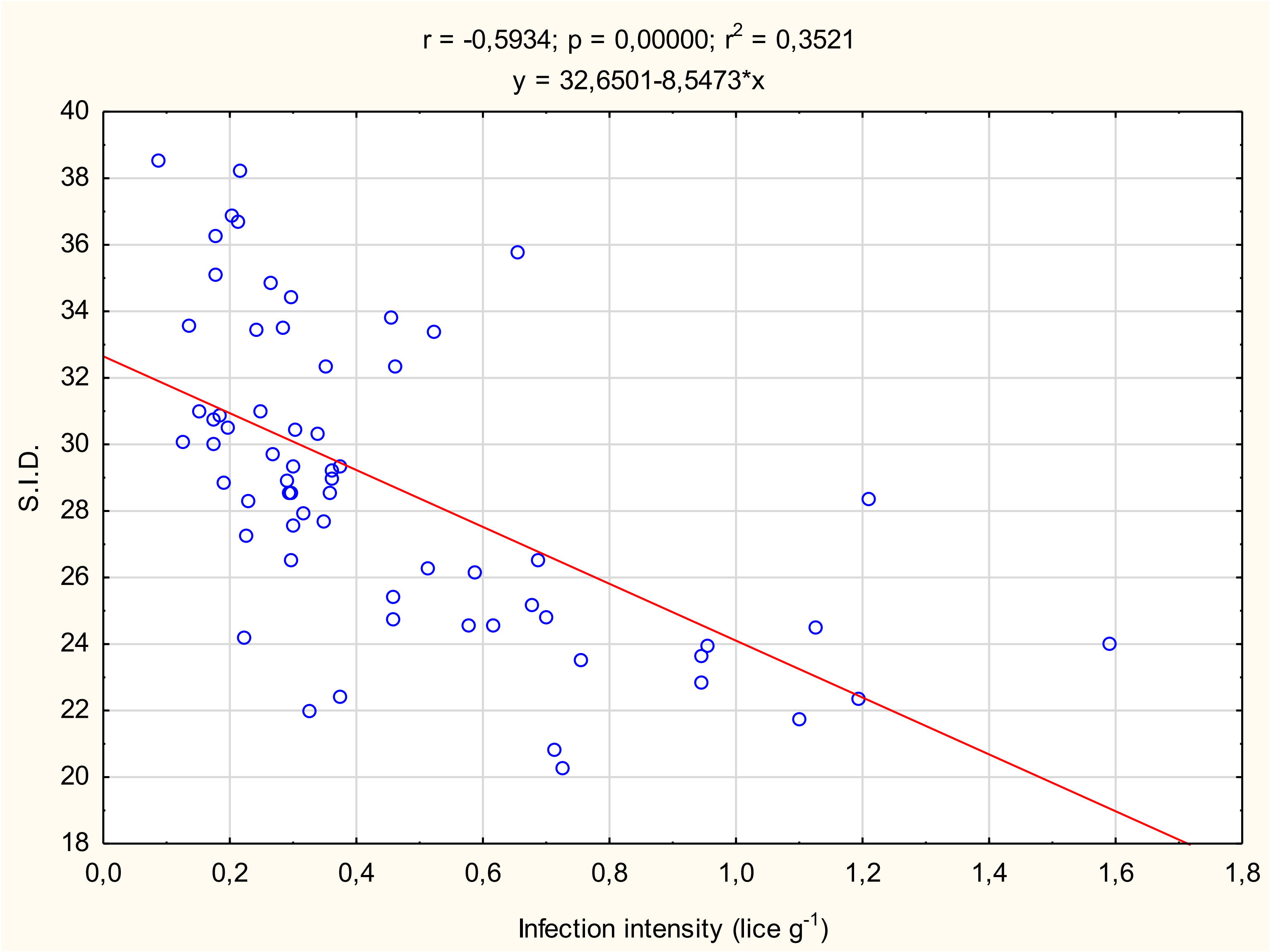

Sup. Fig. 18. Simple linear regression between lice infection intensity (lice g-1) and plasma S.I.D.

Sup. Fig. 19

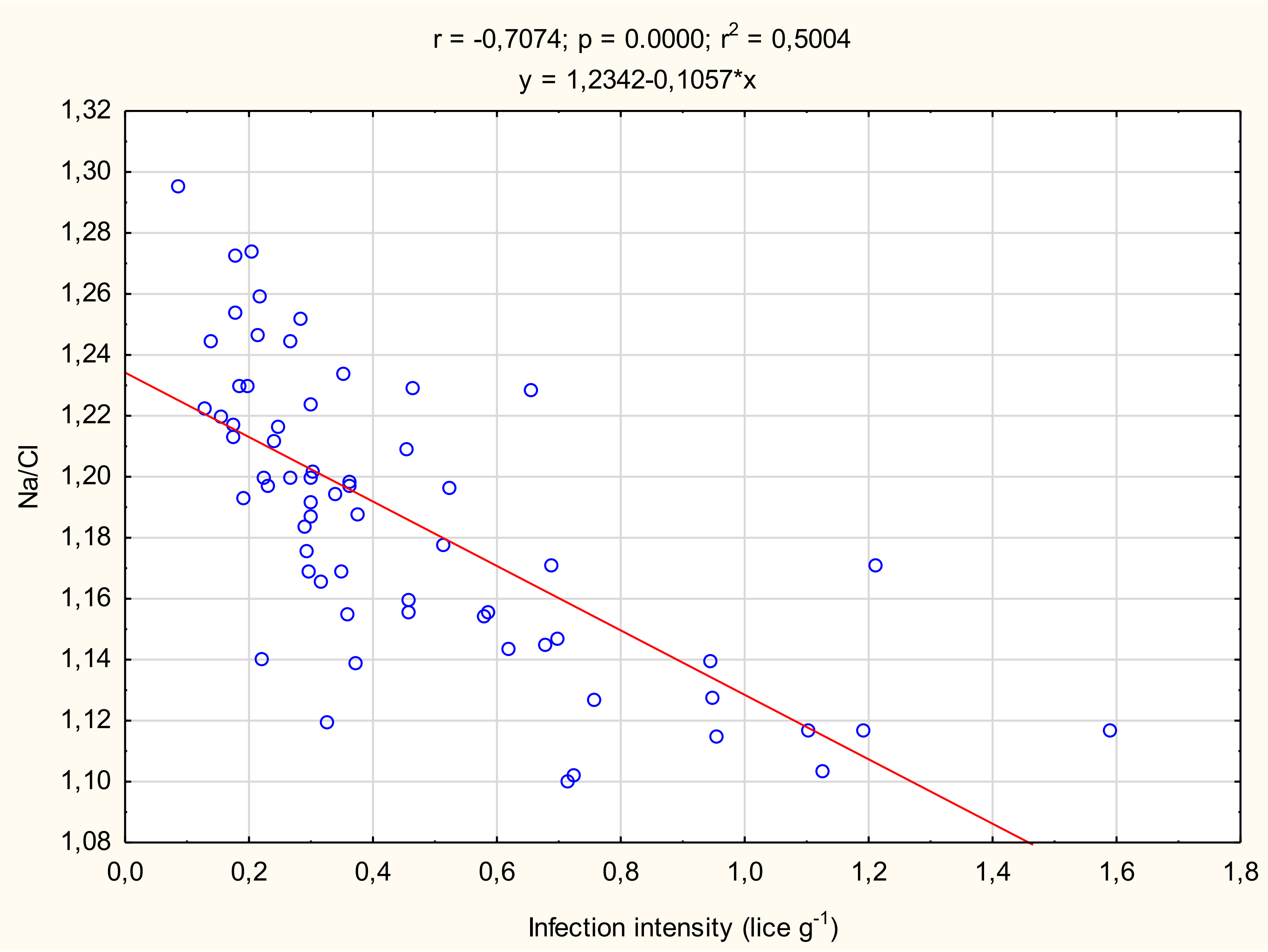

Sup. Fig. 19. Simple linear regression between lice infection intensity (lice g-1) and plasma Na+ / Cl- ratio.

Sup. Table 1. Mean ± SE length (cm), weight (g) and condition factor (CF).

Time point	Parameter	Control			Louse1			Louse2			Louse1+2			P-value	
		IM ♂	M ♂	IM ♀	IM ♂	M ♂	IM ♀	IM ♂	M ♂	IM ♀	IM ♂	M ♂	IM ♀	Phenotype	Treatment
Start 1st infection	Length	19.1±0.5	19.1±0.4	19.5±0.4	18.9±0.4	19.2±0.5	19.1±0.3							0.69173	0.56022
	Weight	101.7±7.4	104.3±7.4	107.1±6.9	96.1±7	108.9±8.7	100.1±4.3							0.50036	0.63610
	CF*	1.41±0.02<br>ab	1.45±0.02<br>ab	1.38±0.01<br>b	1.39±0.02<br>ab	1.47±0.02<br>a	1.42±0.01<br>ab							***0.00239***	0.16153
Finish 1st infection	Length	19.9±0.5	19.9±0.5	20.3±0.4	19.5±0.5	19.9±0.5	19.8±0.3							0.72641	0.38148
	Weight	116.8±8.4	122.4±8.4	123.7±7.8	105.5±7.2	122.2±9.2	110.7±4.6							0.33742	0.25600
	CF*	1.43±0.03<br>b	1.52±0.02<br>a	1.42±0.01<br>b	1.4±0.03<br>b	1.51±0.02<br>a	1.41±0.01<br>b							***0.00003***	0.63399
Start 2nd infection	Length	19.8±0.5	20.6±0.8	20.9±0.3	19.1±0.6	20.4±0.7	19.4±0.3	19.8±0.8	19.7±0.7	20.2±0.3	19.4±0.5	19.4±0.4	20.4±0.3	0.25363	0.21088
	Weight	107.7±9.1	138.1±17.5	126.6±5.5	94.1±10.4	123.4±14.8	98.4±4.6	106.7±17.1	112.8±12.4	114.3±5.8	96.1±8.3	101.8±5.6	114.7±4.6	0.18848	0.12811
	CF*	1.35±0.02<br>abcd	1.46±0.03<br>a	1.35±0.01<br>bcd	1.29±0.02<br>bcd	1.4±0.03<br>abc	1.31±0.01<br>cd	1.29±0.04<br>bcd	1.41±0.02<br>ab	1.35±0.01<br>bcd	1.27±0.02<br>d	1.37±0.02<br>abc	1.33±0.01<br>bcd	***0.00006***	0.22614
Finish 2nd infection	Length	20.9±0.6	21.6±0.9	21.8±0.3	19.8±0.7	21.3±0.7	20.2±0.3	20.4±0.9	20.5±0.6	20.8±0.3	20.1±0.5	20±0.4	21±0.3	0.34457	0.13525
	Weight	117.8±10	148.1±18.9	136.5±6	100.9±13	135.5±17.5	105.2±4.9	110.3±19.5	117.8±13.1	115.6±6.4	98.7±9.5	105.2±5.6	116.1±5.1	0.21064	0.07025
	CF*	1.27±0.03<br>abc	1.36±0.03<br>a	1.28±0.02<br>abc	1.24±0.03<br>abc	1.32±0.03<br>ab	1.24±0.02<br>abc	1.19±0.03<br>bc	1.3±0.02<br>ab	1.24±0.02<br>bc	1.17±0.02<br>c	1.29±0.02<br>abc	1.23±0.02<br>bc	***0.00000***	0.08893
Mid FW follow-up	Length	21.3±0.8	22.3±1.2	22.7±0.5	20.9±1.2	23.1±1.2	20.8±0.5	20.7±0.7	22.1±1.2	21.9±0.5	21.4±1	21.3±0.4	22.2±0.4	0.34956	0.53578
	Weight	131.3±15.8	169.4±29.1	161.4±11.8	124.4±26.7	187.6±31.7	125.3±11.5	119.2±14.8	167.1±26.2	145.8±11.2	132.2±20.7	135.1±5.9	144.8±7.5	0.11476	0.58118
	CF*	1.32±0.03<br>ab	1.43±0.04<br>a	1.32±0.03<br>ab	1.25±0.06<br>b	1.44±0.02<br>a	1.32±0.02<br>ab	1.3±0.03<br>ab	1.45±0.03<br>a	1.34±0.02<br>ab	1.26±0.03<br>b	1.41±0.04<br>ab	1.31±0.02<br>ab	***0.00001***	0.78033
Finish FW follow-up	Length	22.2±0.8	22.7±1.2	23.4±0.5	21.6±1.2	23.3±1.2	21.6±0.6	21.7±0.7	22.5±1.2	22.7±0.5	22.2±1	21.8±0.4	23±0.4	0.51486	0.54796
	Weight	148.8±17.1	165.5±27	179.1±12.1	140.9±28.5	180.6±28.3	140.6±12	145.3±17.6	160.7±24.7	160±11.6	147.2±20.7	137.9±6.2	159.2±8.1	0.59207	0.43310
	CF*	1.33±0.02	1.34±0.03	1.36±0.02	1.29±0.03	1.35±0.02	1.34±0.02	1.39±0.03	1.33±0.03	1.33±0.02	1.27±0.03	1.34±0.03	1.29±0.01	0.50239	0.43013

* At Start 1st infection, infected mature males had significantly higher condition factor compared to control immature females (Suplementary Material 2, Sup. Table 1). At Finish 1st infection (pre-adult lice), mature males had significantly higher condition factor compared to immature males and females within infection groups. At Start 2nd infection, there were no significant differences between the 4 infection groups within the 3 fish phenotypes, while mature males had significantly higher values than immature males and females within the Control and Louse1+2 groups, respectively. This difference had disappeared at Finish 2nd infection. At Mid FW follow-up, however, mature males had significantly higher condition factor compared to immature males within the Louse1 group.
